# The THO complex counteracts TERRA R-loop-mediated telomere fragility in telomerase^+^ cells and telomeric recombination in ALT^+^ cells

**DOI:** 10.1093/nar/gkad448

**Published:** 2023-05-29

**Authors:** Rita Valador Fernandes, Joachim Lingner

**Affiliations:** Swiss Institute for Experimental Cancer Research (ISREC), School of Life Sciences, École Polytechnique Fédérale de Lausanne (EPFL), 1015 Lausanne, Switzerland; Swiss Institute for Experimental Cancer Research (ISREC), School of Life Sciences, École Polytechnique Fédérale de Lausanne (EPFL), 1015 Lausanne, Switzerland

## Abstract

Telomeres are the nucleoprotein structures at the ends of linear chromosomes. Telomeres are transcribed into long non-coding Telomeric Repeat-Containing RNA (TERRA), whose functions rely on its ability to associate with telomeric chromatin. The conserved THO complex (THOC) was previously identified at human telomeres. It links transcription with RNA processing, decreasing the accumulation of co-transcriptional DNA:RNA hybrids throughout the genome. Here, we explore the role of THOC at human telomeres, as a regulator of TERRA localization to chromosome ends. We show that THOC counteracts TERRA association with telomeres via R-loops formed co-transcriptionally and also post-transcriptionally, *in trans*. We demonstrate that THOC binds nucleoplasmic TERRA, and that RNaseH1 loss, which increases telomeric R-loops, promotes THOC occupancy at telomeres. Additionally, we show that THOC counteracts lagging and mainly leading strand telomere fragility, suggesting that TERRA R-loops can interfere with replication fork progression. Finally, we observed that THOC suppresses telomeric sister-chromatid exchange and C-circle accumulation in ALT cancer cells, which maintain telomeres by recombination. Altogether, our findings reveal crucial roles of THOC in telomeric homeostasis through the co- and post-transcriptional regulation of TERRA R-loops.

## Graphical Abstract

**Graphical Abstract:**
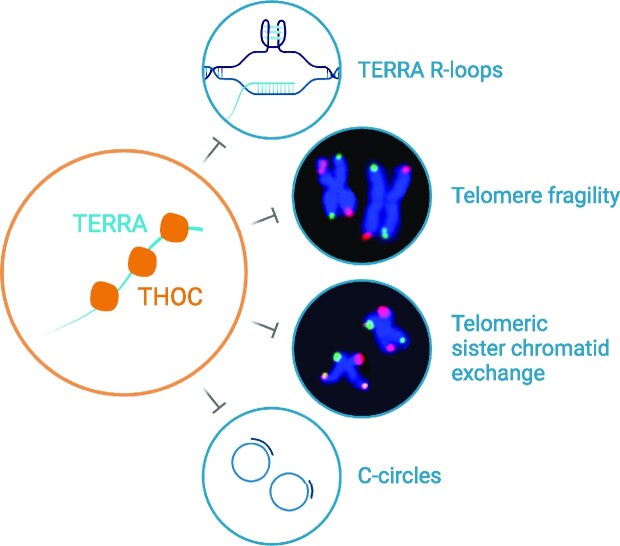

## INTRODUCTION

Telomeres are the nucleoprotein structures at the termini of linear chromosomes. They are crucial for genome stability and ensure that chromosome ends are not inappropriately recognized as sites of DNA damage. Telomere functions at mammalian telomeres are in part achieved by a telomere-specific protein complex termed shelterin. It includes the double strand (ds) DNA-binding proteins TRF1 and TRF2, POT1 which binds the single stranded telomeric overhang, TIN2 and TPP1 which connect POT1 to TRF1 and TRF2, and TRF2-bound Rap1 (1).

Despite the heterochromatic features found at telomeres (2), RNA polymerase II drives transcription of the telomeric long noncoding RNA TERRA—Telomeric Repeat containing RNA, using the C-rich strand as a template (3). In human cells, transcription of TERRA stems from promoters residing within subtelomeric sequences in most chromosome arms, and proceeds through the telomeric repetitive TTAGGG tract (4–6). For that reason, TERRA is a heterogeneous class of lncRNAs, comprising chromosome arm-specific subtelomeric-derived sequences at the 5′ end, followed by a variable number of UUAGGG repeats (6–8).

Several functions regarding telomere maintenance and stability have been attributed to TERRA. Namely, TERRA has been proposed to contribute to the regulation of the heterochromatic structure of telomeres (5,8), particularly when telomeres are depleted of the shelterin protein TRF2—which results in upregulation of TERRA and activation of a DNA damage response (5). In addition, the modulation of telomerase—the ribonucleoprotein which extends the telomeric DNA—by TERRA has previously been proposed. *In vitro*, TERRA was shown to bind the telomerase RNA template, thus acting as an inhibitor of human telomerase (9). On the other hand, *in vivo* experiments in budding yeast suggested that TERRA preferentially guides telomerase to short telomeres, which are then selectively elongated (10). While the majority of cancer cells maintains telomere length by overexpressing telomerase, about 10% of cancer types compensates telomere loss by a mechanism termed alternative lengthening of telomeres (ALT), which relies on homologous recombination (HR) (11). In human ALT cells, TERRA was shown to be a crucial factor involved in telomere maintenance, by promoting a finely-tuned equilibrium of replication stress required for triggering of HR by break-induced replication, allowing telomere elongation (12–15). Also in *Saccharomyces cerevisiae* cells, TERRA was shown to accumulate at critically short telomeres, where it is thought to promote replication stress, resulting in homology-directed repair and telomere elongation (16).

Notably, most functions attributed to TERRA in telomere maintenance were shown to depend on direct DNA:RNA base pair interactions (hybrids), termed R-loops (12,13,16–18). Such structures form when TERRA RNA invades the telomeric dsDNA helix, base pairing with the C-rich telomeric strand, leaving the TTAGGG-containing DNA strand displaced. R-loops were shown to form co-transcriptionally *in cis*—as the transcription machinery progresses through the telomeric tract –, as well as post-transcriptionally *in trans*—at telomeric loci other than the one from which the RNA originated (19). Several factors involved in the regulation of telomeric R-loops have been identified and characterized (reviewed in (20)). Among which, the DNA recombinase RAD51 was shown to be required for the formation of telomeric DNA:RNA hybrids (19). More recently, another HR protein, RAD51AP1, has also been implicated in mediating TERRA R-loops in ALT cancer cells (21,22). On the other hand, factors restraining the accumulation of telomeric R-loops include the shelterin proteins TRF1 and POT1 (23,24), as well as RNase H enzymes (12)—which remove DNA:RNA hybrids through the endonucleolytic cleavage of the engaged RNA molecules.

R-loops have primarily been viewed as harmful by-products of transcription, posing an obstacle for the progression of DNA and RNA polymerases, thus jeopardizing genome stability. However, their involvement in a myriad of cellular processes has been demonstrated (25). At chromosome ends, R-loops are thought to contribute to DNA damage checkpoint signalling when telomeres become damaged or very short (16,17,19). Additionally, as mentioned above, R-loops appear to be a requirement for appropriate telomerase-independent telomere elongation in ALT cancer cells. Altogether, this underscores the relevance behind a comprehensive analysis of the regulation and impact of telomeric R-loops. In this study, we aimed at understanding the roles undertaken by the THO (suppressor of transcription defects of *hpr1Δ* mutants by overexpression) complex at human telomeres.

The THO complex (THOC) was originally identified in *S. cerevisiae* as a functional unit involved in transcriptional elongation of genes with high GC content or that are particularly long, suppressing hyperrecombination events (26–28). In human cells, THOC comprises six subunits: THOC1, -2, -3, -5, -6 and -7 (29). It facilitates transcription of a large number of human genes, and often co-localizes with splicing factors in nuclear speckles (30–32). In addition to its function during transcription, THOC plays a conserved role in RNA nuclear export, as an element of the larger TREX (Transcription and Export) complex, together with the helicase and splicing factor UAP56/DDX39B and the RNA nuclear export protein Aly/ALYREF/THOC4 (along with other factors) (33–35). Remarkably, THOC/TREX have also been described to take part in the regulation of DNA:RNA hybrids formed across the genome during transcription, counteracting the accumulation of R-loop-inducing DNA damage (30,31,36). THOC/TREX factors are thought to be recruited to spliced, 5′ capped RNAs during RNA biogenesis, and contribute to the adequate formation of ribonucleoprotein complexes (32,37–40). Thus, THOC/TREX bridge transcription with RNA processing and nuclear export.

All THOC subunits were previously detected in purified human telomeric chromatin by mass-spectrometry (41). In yeast, several THOC subunits have also been detected at chromosome ends (42). THOC was previously proposed to indirectly counteract telomeric elongation, through the regulation of Rif1 mRNA (a negative regulator of telomere length in *S. cerevisiae*) (43). On the other hand, yeast THOC subunits Hpr1 and Thp2 were demonstrated to counteract TERRA-mediated telomeric R-loops. Genetic experiments further suggested that Thp2 protects chromosome ends from telomere shortening in an R-loop-independent but Exonuclease 1-dependent manner (42). At human telomeres, the roles of the THO complex remained elusive.

Here, we demonstrate that THOC subunits 1 and 2 counteract R-loops formed at human telomeres. Resorting to a previously developed system to detect the nuclear localization of ectopically-expressed TERRA molecules (19), we show that, unexpectedly, the THO complex also counteracts the association of TERRA with telomeres post-transcriptionally, *in trans*, via R-loops. Additionally, we find that the THO complex is recruited to telomeres when cells are depleted of the ribonuclease RNaseH1, which specifically degrades DNA:RNA hybrids. In addition, THOC interacts with nucleoplasmic TERRA. Therefore, we propose that THOC counteracts telomeric R-loops through several mechanisms. First, THOC may prevent the association of TERRA with telomeric DNA through R-loops during transcription. Second, THOC contributes to the resolution of DNA:RNA hybrid structures that have formed post-transcriptionally. Third, through binding of TERRA in the nucleoplasm, THOC may counteract TERRA association with telomeric DNA post-transcriptionally. In addition, we observe that loss of THOC subunits results in fragility at telomeres replicated by leading strand synthesis, and also to a lesser extent by lagging strand synthesis. Finally, in ALT cells—but not in telomerase-positive HeLa cells—loss of THOC leads to an increase in the frequency of telomeric sister-chromatid exchange events and increasing amounts of C-circles, likely as a consequence of accumulated R-loops at the telomeric tract in these cells. Thus, our data demonstrate that THOC promotes telomere stability through the regulation of the lncRNA TERRA.

## MATERIALS AND METHODS

### Cell culture

HeLa, U2OS, Saos2 and Hek293T cell lines were cultured in Dulbecco's modified Eagle's medium (DMEM) (Gibco), supplemented with 10% fetal bovine serum (FBS) and 100 U/ml of penicillin/streptomycin. Cells were maintained in a controlled humidified atmosphere with 5% (v/v) CO_2_, at 37°C. Generation of rtTA^+^ PCP-GFP^+^ HeLa cells for PP7-15qTERRA expression was previously described (19). Generation of HeLa cells with ca. 30 kb average telomere length was previously described (44). Suspension Hek293E cells were cultured in EX-CELL 293 Serum-Free Medium (Merck), supplemented with 4 mM GlutaMAX (Thermo Fisher Scientific) with 5% (v/v) CO_2_, at 37°C, with constant agitation.

### siRNA and plasmid transfection

180 000 cells/well were plated in six-well plates one day before siRNA transfection. The following day, growth medium was replaced by antibiotic-free DMEM supplemented with 10% FBS, and cells were transfected with 20 pmol siRNA (see Supplementary Table S1) using calcium phosphate transfection. The following day, antibiotic-supplemented growth medium was replenished, and cells were transfected with Lipofectamine 2000 (Invitrogen) using 2.5 μg plasmid DNA (see Supplementary Table S2), following manufacturer's instructions. When doxycycline-inducible constructs were used, 1 μg/ml doxycycline was added to the medium 24 h after plasmid DNA transfection. Cells were harvested 72 h post-siRNA transfection and 48 h post-plasmid DNA transfection.

### Lentivirus production and cell transduction

Hek293T cells were plated one day before transfection for lentivirus production. The following day, growth medium was replaced by Opti-MEM (Thermo Fisher Scientific). Cells were transfected with 1 μg pMD2.G plasmid, 3 μg pCMVR8.74 plasmid and 4 μg of lentivirus transfer plasmid (see Supplementary Table S2), using Lipofectamine 2000 (Invitrogen), following manufacturer's instructions. Cells were kept in the transfection mix overnight, which was replaced by antibiotic-supplemented growth medium the next day. Supernatants containing lentiviral particles were collected on the two following days, and filtered through syringes with 0.45 μm filter units. Viruses were aliquoted and kept at –80°C. 1 ml of lentivirus-containing medium was carefully added to each 6 cm dish containing recipient cells plated one day before transduction. Cells were split into 15 cm dishes, and selection was initiated with appropriate antibiotic 48 h post-transduction. Puromycin selection was done at 1 μg/ml final concentration.

### Immunofluorescence and telomeric fluorescence *in situ* hybridization (FISH)

Cells were grown on round coverslips. Coverslips were washed twice in 1× PBS, fixed with 4% paraformaldehyde in PBS for 10 min at room temperature, and washed twice in 1× PBS. Coverslips were then incubated in detergent solution (0.1% Triton X-100, 0.02% SDS in 1× PBS) for 5 min, and incubated in 2% BSA dissolved in PBS for 10 min. Incubation with primary antibody (see Supplementary Table S3) in blocking solution (10% normal goat serum in 2% BSA dissolved in PBS) was done overnight at 4°C, in a humidified chamber. The following day, three washes with 2% BSA dissolved in PBS were done for 4 min/wash, before incubation with secondary antibody (see Supplementary Table S3) in blocking solution, for 30 min at room temperature, in a humidified chamber. Coverslips were then washed three times in 1× PBS, fixed with 4% paraformaldehyde in PBS for 5 min at room temperature, washed three times in 1× PBS, and dehydrated with 70%, 95% and 100% ethanol.

For FISH staining, hybridization was done with 100 nM Cy3-[CCCTAA]_3_ PNA probe (PNA Bio) in 15 μl hybridization mix per coverslip (10 mM Tris pH 7.4, 70% formamide, 0.5% blocking reagent (Roche)), at 80°C for 3 min, followed by 3 h at room temperature, in a humidified chamber. Slides were washed twice with wash buffer 1 (10 mM Tris pH 7.4, 70% formamide) for 15 min/wash, and then washed three times with wash buffer 2 (0.1 M Tris pH 7.4, 0.15 M NaCl, 0.08% Tween-20) for 5 min/wash. DAPI was added to the second last wash at 0.1 μg/ml. Slides were dehydrated with 70%, 95% and 100% ethanol, air-dried and mounted with Vectashield.

Images were acquired with an Upright Zeiss Axioplan equipped with a 100×/1.40 oil objective.

### Telomeric FISH on metaphase chromosomes

Cells were treated with 0.05 μg/ml demecolcine for 2 h before harvesting by trypsinization, resuspended in hypotonic solution (0.056 M KCl), and incubated at 37°C for 7 min. Cells were fixed in cold methanol:glacial acetic acid (3:1) solution overnight at 4°C. Fixed cells were dropped onto glass slides, incubated at 70°C for 1 min in a humidified oven, and air-dried overnight at room temperature. Slides were incubated in 4% formaldehyde in PBS for 5 min, washed three times in 1× PBS and dehydrated with 70%, 95% and 100% ethanol. Hybridization was done with 100 nM Cy3-[CCCTAA]_3_ PNA probe (PNA Bio) in 70 μl hybridization mix (10 mM Tris pH 7.4, 70% formamide, 0.5% blocking reagent (Roche)), at 80°C for 3 min, followed by 3 h at room temperature, in a humidified chamber. Slides were washed twice with wash buffer 1 (10 mM Tris pH 7.4, 70% formamide) for 15 min/wash, and then washed three times with wash buffer 2 (0.1 M Tris pH 7.4, 0.15 M NaCl, 0.08% Tween-20) for 5 min/wash. DAPI was added to the second last wash at 0.1 μg/ml. Slides were dehydrated with 70%, 95% and 100% ethanol, air-dried and mounted with Vectashield.

Images were acquired with an Upright Zeiss Axioplan equipped with a 100×/1.40 oil objective, or with a Leica SP8 confocal microscope equipped with a 63×/1.40 oil objective and a DFC 7000 GT camera.

### Chromosome orientation (CO)-FISH on metaphase chromosomes

CO-FISH staining was performed as previously described (23), with some modifications. HeLa or U2OS cells were incubated with BrdU/BrdC (3:1 at a final concentration of 10 μM) for 15.5 or 17.5 h, and with 0.1 or 0.2 μg/ml demecolcine for 2 or 5 h before harvesting by trypsinization, respectively, resuspended in hypotonic solution (0.056 M KCl), and incubated at 37°C for 7 min. Cells were fixed in cold methanol:glacial acetic acid (3:1) solution overnight at 4°C. Fixed cells were dropped onto glass slides, incubated at 70°C for 1 min in a humidified oven, and air-dried overnight at room temperature. Slides were re-hydrated in 1× PBS for 5 min and treated with 250 μg/ml RNaseA (Promega) for 1 h at 37°C. Slides were washed in 1× PBS for 2 min and incubated with 10 μg/ml Hoechst 33258 (Invitrogen) in 1×PBS for 15 min at room temperature. Slides were then exposed to 365 nm UV light using a Stratagene Stratalinker 1800 UV irradiator set to 5400 J, covered by minimal amount of 1× PBS. A brief wash in H_2_O was done after irradiation, followed by treatment with 10 U/μl Exonuclease III (New England Biolabs) for 1 h at 37°C. Slides were incubated in 4% formaldehyde in PBS for 3 min, washed three times in 1× PBS, and dehydrated with 70%, 95% and 100% ethanol. Following overnight air-drying of slides, hybridizations were done sequentially using 0.22 μM TYE563- or 6-FAM-labeled LNA probes (Qiagen) (TeloC LNA probe: 5′ TYE563-CCC*TAACCC*TAACCC*TAA 3′; TeloG LNA probe: 5′ 6-FAM-T*TAGGGT*TAGGGT*TAGGG—where asterisks (*T) indicate LNA nucleotides) in 60 μl hybridization mix per slide (2× SSC, 30% formamide, 0.5% blocking reagent (Roche)), at room temperature for 2 h each. After each hybridization, slides were washed three times in 2× SCC for 3 min and dehydrated with 70%, 95% and 100% ethanol. After the last probe hybridization, DAPI was added to the second 2xSSC wash at 0.1 μg/ml. Slides were air-dried and mounted with ProLong Diamond Antifade mountant.

Images were acquired with a Leica SP8 confocal microscope equipped with a 63×/1.40 oil objective and a DFC 7000 GT camera.

### Western blotting

Cells were collected by trypsinization, washed with 1× PBS and resuspended in 2× Laemmli buffer, to a final concentration of 10 000 cells/μl of Laemmli buffer. Samples were incubated at 95°C for 5 min. Proteins were separated on a 4–15% SDS-PAGE precast gel (Mini-PROTEAN TGX Gels, Bio-Rad) and transferred onto a 0.2 μm nitrocellulose membrane (Amersham). Membranes were blocked with blocking solution (3% BSA (w/v) in 1× PBS with 0.1% Tween-20) for 1 h at room temperature and incubated with primary antibodies (see Supplementary Table S3) at 4°C overnight. Membranes were washed three times for 15 min/wash with 1 × PBS with 0.1% Tween-20, and incubated with Horseradish Peroxidase-conjugated secondary antibodies (see Supplementary Table S3) in blocking solution, for 1 h at room temperature. Membranes were then washed three times for 5 min/wash with 1 × PBS with 0.1% Tween-20. ChemiGlow Chemiluminescence Substrate (Bio Techne) was used to develop the signal, which was detected by a Fusion FX imaging system (Vilber).

### RNA isolation, RNA dot blot and RT-qPCR

RNA was isolated from 3 to 5 million cells with the NucleoSpin RNA kit (Macherey-Nagel). Two on-column and one in-solution rDNase digestions (Macherey-Nagel) were performed.

For RNA dot blot, purified RNA was digested with RNase (DNase-free, Roche), as a control. All samples were denatured at 65°C for 3 min and blotted onto a Hybond-XL membrane (Amersham) using a dot-blot apparatus (Bio-Rad). RNA was UV-crosslinked to the membrane. The membrane was then blocked in Church buffer (0.5 M NaHPO_4_, 1 mM EDTA, pH 8.0, 1% (w/v) BSA, 7% SDS) for at least 1 h at 50°C, and hybridized with a ^32^P-radiolabeled telomeric probe in Church buffer at 50°C overnight. The membrane was washed twice in 2x SSC, 0.5% SDS and twice in 1× SSC, 0.5% SDS, for 15 min/wash. The membrane was exposed to a phosphorimager screen. Radioactive signal was detected with a Typhoon Biomolecular Imager (GE). After signal detection, the membrane was stripped by incubation with boiled 0.1× SCC, 1% SDS at 55°C, three times for 30 min each, and blocked in Church buffer for at least 1 h at 55°C. A ^32^P-radiolabeled 18S rRNA oligonucleotide probe was used for hybridization at 55°C overnight. The membrane was then treated as described for the telomeric probe.

For RT-qPCR analysis of TERRA levels, 3 μg of RNA were reverse-transcribed using 200 U of SuperScript III reverse transcriptase (Thermo Fisher Scientific), GAPDH and TERRA reverse primers (see Supplementary Table S4). Reverse transcription was performed at 55°C for 1 h, followed by heat inactivation at 70°C for 15 min. Reactions without reverse transcriptase were included as a control. 5% of the reaction were mixed with 2 × Power SYBR Green PCR Master Mix (Thermo Fisher Scientific) and 0.5 μM forward and reverse qPCR primers (see Supplementary Table S4). qPCR was carried out at 95°C for 10 min, followed by 95°C for 15 sec, and annealing and extension at 60°C for 1 min for 40 cycles in a QuantStudio 6 Flex Real-Time PCR system (Thermo Fisher Scientific).

### RNA immunoprecipitation (RNA-IP)

Cells were harvested by trypsinization (or collected by centrifugation in the case of suspension Hek293E cells), counted, washed with 1× PBS and placed on ice. Cells were then lysed in RLN buffer (50 mM Tris–HCl pH 8.0, 140 mM NaCl, 1.5 mM MgCl_2_, 0.5% NP-40, 1 mM dithiothreitol (DTT)), supplemented with 400 U/ml RNasin Plus (Promega) and a protease inhibitor cocktail (cOmplete, Roche) (100 × 10^6^ cells/ml of RLN buffer). Lysates were homogenized with a Dounce homogenizer, incubated on a rotating wheel for 20 min at 4°C, and centrifuged at high-speed for 10 min. The supernatant was collected and pre-cleared with magnetic protein G Dynabeads (Thermo Fisher Scientific) on a rotating wheel for 1 h at 4°C (70 μl beads per 1 ml of extract). Pre-cleared extracts equivalent to 50 × 10^6^ cells were used per immunoprecipitation with 6 μg of antibody (see Supplementary Table S3), and incubated on a rotating wheel for 2 hours at 4°C. Input samples equivalent to 10% of each immunoprecipitation were also collected. 35 μl of magnetic protein G Dynabeads (Thermo Fisher Scientific) pre-blocked with yeast transfer RNA were then added to each immunoprecipitation sample and incubated on a rotating wheel overnight at 4°C. Samples were then washed at 4°C for 5 min/wash on a rotating wheel with RLN buffer supplemented with 6 mM EDTA pH 8.0, 0.5% NP-40 and 20 U/ml SUPERase In RNase inhibitor (Thermo Fisher Scientific). RNA was eluted from beads with 1500 rpm agitation, in 1% SDS, 5 mM EDTA pH 8.0 and 5 mM 2-mercaptoethanol, at 42°C for 30 min (for input and immunoprecipitation samples), followed by 65°C for 30 min (for immunoprecipitation samples only). The RNA was purified following the RNA clean-up protocol of the NucleoSpin RNA isolation kit (Macherey-Nagel) and eluted in 100 μl H_2_O. Half of each immunoprecipitation sample was digested with RNase (DNase-free, Roche)). Input samples were diluted appropriately to ensure that the amount of probed RNA in each immunoprecipitated sample lies within the linear dynamic range of the assay. RNA dot blot was done as described above.

### Chromatin immunoprecipitation (ChIP)

Cells were harvested by trypsinization 72 h post-siRNA transfection, counted, and washed with cold 1× PBS. For Western blot analysis of depletions, ca. 0.5 × 10^6^ cells/condition were collected. For ChIP, 1 × 10^7^ cells/condition were crosslinked in 1 ml 1% methanol-free formaldehyde in PBS, for 15 min, on a rotating wheel, at room temperature. Formaldehyde was quenched by adding 250 mM Tris pH 8.0 in PBS, and incubating for 5 min, on a rotating wheel, at room temperature. Cells were then washed three times with cold PBS and kept at 4°C throughout the procedure until the crosslink reversal stage. Cells were washed once with 1 ml LB3 buffer (10 mM Tris pH 8.0, 200 mM NaCl, 1 mM EDTA, 0.5 mM EGTA, 0.1% Na-Deoxycholate, 0.25% sodium lauroyl sarkosinate, supplemented with protease inhibitor cocktail (cOmplete, Roche)), resuspend in 1 ml LB3 buffer, transferred to sonication vials with AFA fiber (Covaris), and sonicated with a Focused-Ultrasonicator (E220, Covaris) (10% duty factor, 140 W power, 200 cycles per burst, for 20 min), to achieve fragments of < 500 bp. Sonicated samples were centrifuged at 21000 g for 15 min. Supernatants were collected and diluted 1:2 in IP dilution buffer (16.7 mM Tris pH 8.0, 1.1% Triton-X, 300 mM NaCl, 1.2 mM EDTA pH 8.0, supplemented with protease inhibitor cocktail (cOmplete, Roche)). Samples were pre-cleared with sepharose protein G beads (Cytiva) pre-blocked with yeast transfer RNA, on a rotating wheel for 1 h, at 4°C. Pre-cleared extracts equivalent to 2 × 10^6^ cells were used per immunoprecipitation with 4 μg of antibody (see Supplementary Table S3), and 20 μl sepharose protein G beads pre-blocked with yeast transfer RNA, on a rotating wheel, at 4°C, overnight. Input samples equivalent to 10% of each immunoprecipitation were also collected. The following day, samples were washed at 4°C for 5 min/wash on a rotating wheel with buffer Wash 1 (0.1% SDS, 1% Triton, 2 mM EDTA pH 8.0, 20 mM Tris pH 8.0, 300 mM NaCl), buffer Wash 2 (0.1% SDS, 1% Triton, 2 mM EDTA pH 8.0, 20 mM Tris pH 8.0, 500 mM NaCl), buffer Wash 3 (250 mM LiCl, 1% NP-40, 1% Na-deoxycholate, 1 mM EDTA pH 8.0, 10 mM Tris pH 8.0) and TE buffer (1 mM EDTA pH 8.0, 10 mM Tris pH 8.0). Washed beads and input samples were resuspended in crosslink reversal buffer (0.1% SDS, 0.1 M sodium bicarbonate, 0.5 mM EDTA pH 8.0, 20 mM Tris pH 8.0, supplemented with 10 μg/ml RNase (DNase-free (Roche)) at 65°C, on a rotating wheel, overnight. DNA was isolated with the NucleoSpin Gel and PCR Clean-up kit with NTB buffer (Macherey-Nagel) and eluted in 40 μl H_2_O. Samples were then analyzed by dot blot (see below).

### DNA:RNA immunoprecipitation (DRIP)

DRIP was performed as previously described (45). Cells were harvested by trypsinization 72 h post-siRNA transfection and 48 h post-plasmid DNA transfection, counted and washed with cold 1× PBS. For Western blot analysis of depletions, ca. 0.5 × 10^6^ cells/condition were collected. For DRIP, 1 × 10^7^ cells/condition were resuspended in 175 μl of ice-cold RLN buffer (50 mM Tris–HCl pH 8.0, 140 mM NaCl, 1.5 mM MgCl_2_, 0.5% NP-40, 1 mM dithiothreitol (DTT), and 100 U/ml RNasin Plus (Promega)), incubated on ice for 5 min, and centrifuged at 300 g for 2 min at 4°C. Nuclei were brought to room temperature and lysed with 500 μl RA1 buffer (NucleoSpin RNA purification kit, Macherey-Nagel) containing 1% 2-mercaptoethanol, and homogenized with a syringe with a 0.9 × 40 mm needle. Nucleic acid extracts were then loaded in Phase Lock Gel heavy (5PRIME) tubes, mixed with 250 μl H_2_O and 750 μl phenol-chloroform-isoamylalcohol (25:24:1) with a pH of 7.8 to 8.2, and centrifuged at 13 000 g for 5 min at room temperature. The aqueous phase was transferred into a new tube. 750 μl cold isopropanol and NaCl to 50 mM were added to the aqueous phase, mixed thoroughly, and incubated on ice for 30 min. Samples were centrifuged at 10 000 g for 30 min at 4°C to precipitate nucleic acids, followed by two washes with 70% cold ethanol. After air-drying, nucleic acids were dissolved in 130 μl of H_2_O, and sonicated with a Focused-Ultrasonicator (E220, Covaris) (10% duty factor, 140 W power, 200 cycles per burst, for 150 s, with an AFA intensifier), to achieve fragments of 100–300 bp. The concentration of fragmented nucleic acids was determined by spectrophotometry with a NanoDrop (ThermoFisher). Appropriate amount (see below) of nucleic acids was digested with 10 μl RNaseH (1 U/μl, Roche)—as a negative control—or H_2_O, in 15 μl RNaseH buffer (20 mM HEPES–KOH pH 7.5, 50 mM NaCl, 10 mM MgCl_2_, 1 mM DTT), in a total volume of 150 μl, and incubated at 37°C for 90 min. Digestion was stopped with 2 μl 0.5 M EDTA (pH 8.0) per sample. Samples were diluted 1:10 in DIP-1 buffer (10 mM HEPES–KOH pH 7.5, 275 mM NaCl, 0.1% Na-deoxycholate, 0.1% SDS, 1% Triton X-100) and pre-cleared with 40 μl of sepharose protein G beads (Cytiva) for 1 h, on a rotating wheel, at 4°C. 30 μg of diluted nucleic acids from HeLa cells or 12.5 μg of diluted nucleic acids from U2OS cells were used per immunoprecipitation with 3 μg (HeLa) or 6 μg (U2OS) of S9.6 antibody (Kerafast) or mouse IgG antibody (see Supplementary Table S3), and 20 μl of sepharose protein G beads (Cytiva), and incubated on a rotating wheel at 4°C overnight. Nucleic acids equivalent to 1% of each immunoprecipitation sample were collected as input. The following day, samples were washed at 4°C for 5 min/wash on a rotating wheel with buffer DIP-2 (50 mM HEPES–KOH pH 7.5, 140 mM NaCl, 1 mM EDTA pH 8.0, 1% Triton X-100, 0.1% Na-deoxycholate), buffer DIP-3 (50 mM HEPES–KOH pH 7.5, 500 mM NaCl, 1 mM EDTA pH 8.0, 1% Triton-X100, 0.1% Na-deoxycholate), buffer DIP-4 (10 mM Tris–HCl pH 8.0, 1 mM EDTA pH 8.0, 250 mM LiCl, 1% NP-40, 1% Na-deoxycholate), and TE buffer (10 mM Tris–HCl pH 8.0, 1 mM EDTA pH 8.0). Immunoprecipitation and input samples were resuspended overnight at 65°C with 100 μl elution buffer (20 mM Tris–HCl pH 8.0, 0.1% SDS, 0.1 M NaHCO_3_, 0.5 mM EDTA pH 8.0) containing 10 μg/ml RNase (DNase-free (Roche)). DNA was isolated with the QIAquick PCR Purification kit (Qiagen) and eluted in 100 μl H_2_O. Samples were then analysed by dot blot or qPCR (see below).

### qPCR analysis of DRIP samples

Each qPCR comprised 1 μl of purified DNA (immunoprecipitation and diluted input samples—as aforementioned), 5 μl Power SYBR Green PCR Master Mix (Thermo Fisher Scientific), 1 μM forward and reverse primers (see Supplementary Table S4), and H_2_O up to 10 μl total reaction volume. Each input or immunoprecipitation sample was run in technical duplicate. qPCR was caried at 95°C for 10 min, followed by 95°C for 15 s, and annealing and extension at 60°C for 1 min for 40 cycles in a QuantStudio 6 Flex Real-Time PCR system (Thermo Fisher Scientific). At least two serial dilutions (with dilution factors of 5 and 50) of each input sample were included, in order to perform a regression analysis to determine the equation of the standard curve of each input. Using each input equation, the corresponding S9.6 and IgG immunoprecipitations were calculated as percentage of input.

### Dot blot analysis of ChIP and DRIP samples

Input samples (ChIP and DRIP) were diluted appropriately to ensure that the amount of probed DNA in each immunoprecipitated sample was within the linear dynamic range of each assay. Purified DNA (diluted inputs and immunoprecipitated samples) was incubated at 95°C for 5 min and kept on ice. Samples were blotted onto a Hybond-XL membrane (Amersham) using a dot blot apparatus (Bio-Rad), and DNA was UV-crosslinked to the membrane. The membrane was denatured in 0.5 M NaOH, 1.5 M NaCl for 15 min on a shaker at room temperature, neutralized in 0.5 M Tris–Cl pH 7.0, 1.5 M NaCl for 10 min on a shaker at room temperature, and then blocked in Church buffer (0.5 M NaHPO_4_, 1 mM EDTA, pH 8.0, 1% (w/v) BSA, 7% SDS) for at least 1 h at 65°C. The membrane was hybridized with a ^32^P-radiolabeled TeloC probe in Church buffer at 65°C overnight. Washes were done three times for 30 min/wash at 65°C, in 1× SSC, 0.5% SDS. The membrane was then exposed to a phosphorimager screen. Radioactive signal was detected with a Typhoon Biomolecular Imager (GE).

### C-circle assay

The C-circle assay was performed as previously described (46). Briefly, genomic DNA was extracted with phenol-chloroform-isoamyl alcohol (25:24:1) and digested with HinfI and RsaI (NEB) in CutSmart buffer (NEB), overnight at 37°C. 30 ng of digested DNA were incubated with 7.5 U Phi29 DNA polymerase (NEB) (or in its absence for Phi29^−^ control reaction), in the presence of dATP, dTTP and dGTP (1 mM each) at 30°C for 8 h, followed by heat-inactivation at 65°C for 20 min. Reaction products were blotted onto a Hybond-XL membrane (Amersham) using a dot blot apparatus (Bio-Rad), and DNA was UV-crosslinked to the membrane. The non-denatured membrane was blocked in Church buffer (described above) for at least 1 h at 42°C. The membrane was hybridized with a ^32^P-radiolabeled TeloC oligonucleotide probe in Church buffer at 42°C overnight. Washes were done three times for 30 min/wash at 42°C, in 1× SSC, 0.5% SDS. The membrane was then exposed to a phosphorimager screen. Radioactive signal was detected with a Typhoon Biomolecular Imager (GE). For detection of the Phi29^−^ signal, the membrane was stripped and denatured as mentioned above, blocked in Church buffer for at least 1 h at 42°C, and re-probed with a ^32^P-radiolabeled TeloC oligonucleotide probe in Church buffer at 42°C overnight. Washes and signal detection were done as described.

### Telomere restriction fragment length analysis (TRF)

Genomic DNA (gDNA) was extracted with phenol-chloroform-isoamyl alcohol (25:24:1) and digested with HinfI and RsaI (NEB) in CutSmart buffer (NEB), overnight at 37°C. 0.5–2 μg of digested gDNA/lane were resolved by constant-field gel electrophoresis in 0.8% agarose gel in 1× TBE for 17 h at 70 V, or by pulsed-field gel electrophoresis in 1% agarose gel in 0.5x TBE for 16 h at 14°C, at 5.2 V/cm with 0.5 s initial switch and 6 s final switch, using a CHEF-DRII apparatus (Bio-Rad). The gel was dried for 4–5 h at 50°C, denatured in 0.5 M NaOH, 1.5 M NaCl for 15 min on a shaker at room temperature, neutralized in 0.5 M Tris-Cl pH 7.0, 1.5 M NaCl for 10 min on a shaker at room temperature, and then blocked in Church buffer (as described above) for at least 1 h at 50°C. The gel was hybridized with a ^32^P-radiolabeled TeloC probe in Church buffer at 50°C overnight. Washes were done for 1 h/wash at 50°C, in 4× SSC, followed by 4× SSC 0.1% SDS and 2× SSC 0.1% SDS. The gel was then exposed to a phosphorimager screen. Radioactive signal was detected with a Typhoon Biomolecular Imager (GE).

### Software

Images were processed and analysed with ImageJ (2.0.0-rc-69/1.53k). Dot blots were analysed using Aida Image Analyzer (v. 5.1). Preparation of graphs and statistical analyses were performed using GraphPad Prism (v. 9.4.1 (458)). Illustrations were generated with BioRender.

## RESULTS

### The THO complex counteracts telomeric DNA:RNA hybrids

**Figure 1.**
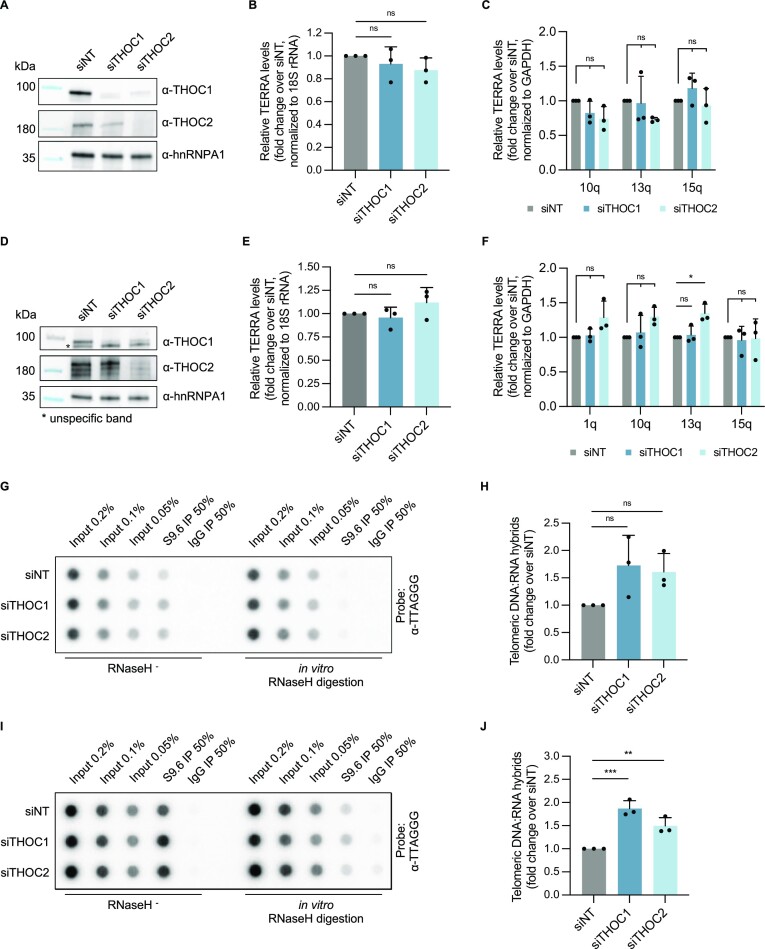
THOC counteracts TERRA R-loops. (**A**) Western blot analysis of depletion efficiency with indicated siRNAs in HeLa cells with 10 kb average telomere length. (B, C) Quantification of TERRA levels in RNA extracts from HeLa cells, determined by RNA dot blot, normalized to 18S rRNA levels (**B**), or by RT-qPCR with indicated TERRA subtelomeric primers, normalized to GAPDH RNA levels (**C**). Data are plotted as fold change over control siNT, and represent mean ± s.d., from three independent biological replicates. One-way analysis of variance (ANOVA) with Dunnett's multiple comparisons test was applied: ns indicates non-significance (*P* > 0.05). (**D**) Western blot analysis of depletion efficiency with indicated siRNAs in U2OS cells. (E, F) Quantification of TERRA levels in RNA extracts from U2OS cells, determined by RNA dot blot, normalized to 18S rRNA levels (**E**), or by RT-qPCR with indicated TERRA subtelomeric primers, normalized to GAPDH RNA levels (**F**). Data are plotted as fold change over control siNT, and represent mean ± s.d., from three independent biological replicates. One-way analysis of variance (ANOVA) with Dunnett's multiple comparisons test was applied: * *P* ≤ 0.05, ns indicates non-significance (*P* > 0.05). (**G**) DRIP assay using anti-DNA:RNA hybrid S9.6 antibody was performed in extracts from HeLa cells. *In vitro* digestion with RNaseH prior immunoprecipitation served as a negative control. All immunoprecipitates and input samples were treated with RNase (DNase-free) and analyzed by DNA dot blot probed with a ^32^P-radiolabelled [CCCTAA]_3_ probe. (**H**) Quantification of telomeric DNA:RNA hybrids in HeLa cell extracts (as in G), as fold change over siNT. Data represent mean ± s.d., from three independent biological replicates. One-way analysis of variance (ANOVA) with Dunnett's multiple comparisons test was applied: ns indicates non-significance (*P* > 0.05). (**I**) DRIP assay using anti-DNA:RNA hybrid S9.6 antibody was performed in extracts from U2OS cells. *In vitro* digestion with RNaseH1 prior immunoprecipitation served as a negative control. All immunoprecipitates and input samples were treated with RNase (DNase-free) and analysed by DNA dot blot probed with a ^32^P-radiolabelled [CCCTAA]_3_ probe. (**J**) Quantification of telomeric DNA:RNA hybrids in U2OS cell extracts (as in I), as fold change over siNT. Data represent mean ± s.d., from three independent biological replicates. One-way analysis of variance (ANOVA) with Dunnett's multiple comparisons test was applied: *** *P* ≤ 0.001, ** *P* ≤ 0.01.

THOC was previously shown to be necessary for appropriate expression of several genes in yeast and human cells, promoting transcriptional elongation by RNA polymerase II (27,30,31,47,48). To understand if THOC is involved in the transcriptional regulation of TERRA affecting its expression levels, we knocked down THOC core subunits 1 and 2 in HeLa cells (Figure 1A). Of note, siRNA mediated targeting of THOC1 caused a reduction of THOC2 and vice versa, indicating that THOC subunits THOC1 and THOC2 are stabilizing one another. We evaluated total TERRA levels by RNA dot blot, probed with a ^32^P-radiolabeled [CCCTAA]_3_ probe and normalized the signal to 18S rRNA (Supplementary Figure S1A; Figure 1B), and by RT-qPCR (Figure 1C). No changes were detected in UUAGGG-containing RNA levels upon depletion of THOC (Figure 1B-C). This indicates that the expression of TERRA is not directly modulated by THOC, nor is the telomeric transcriptional elongation affected by loss of THOC subunits, since a defect in TERRA transcriptional elongation would be reflected in changes in the detected UUAGGG content.

In U2OS cells, which do not use telomerase for telomere length maintenance, but resort instead to the recombination-based ALT pathway, basal TERRA levels are elevated, relative to HeLa or other telomerase-positive cell lines (49). Nonetheless, depletion of THOC subunits in U2OS cells (Figure 1D) did not lead to any perceptible changes in TERRA levels (Supplementary Figure S1B; Figure 1E and F), similarly to what we observed in HeLa cells.

Telomeres are prone to form DNA:RNA hybrids with TERRA (12,16,19). Since THOC has been implicated in counteracting DNA:RNA hybrids across the genome (30,31), we analyzed telomeric DNA:RNA hybrid levels under depletion of THOC. We implemented DNA:RNA immunoprecipitation (DRIP), which uses the R-loop-recognizing S9.6 antibody (50), and quantified the DNA component of the hybrids by DNA dot blot, probed with a C-rich telomeric probe. Nucleic acids were *in vitro* treated with the ribonuclease RNaseH (which specifically hydrolyses the RNA moiety of DNA:RNA hybrids (51)) before the immunoprecipitation step, as a specificity control. Interestingly, depletion of THOC led to a perceptible (yet non statistically significant) increase in telomeric R-loop levels in HeLa cells (Figure 1G and H). In U2OS cells, basal telomeric R-loops were shown to accumulate more frequently, compared to telomerase-positive cells (12). When examining U2OS cells by DRIP dot blot, we observed that loss of THOC in these cells significantly increased the occurrence of telomeric R-loops (Figure 1I and J). This indicates that the THO complex plays a role at human telomeres in counteracting TERRA-mediated R-loops.

Of note, no changes in the average telomere length were detected by terminal restriction fragment analysis on Southern blots, following THOC1 or THOC2 depletion in HeLa, U2OS or Saos2 cells for 72 h (Supplementary Figure S1C–E).

### The THO complex counteracts PP7-15qTERRA associations with telomeres formed *in trans*

While most R-loops are thought to occur only during transcription, TERRA R-loops can also form post-transcriptionally, in dependency of the RAD51 recombinase (19). To investigate putative roles of THOC in the post-transcriptional regulation of TERRA, we used a previously developed system in which TERRA is expressed from plasmids, allowing the characterization of the subnuclear localization of ectopically transcribed TERRA-like molecules (19). It resorts to the inducible-expression of a chimeric RNA molecule comprised of about ninety UUAGGG repeats at the 3′ end, preceded by the 15q subtelomeric-derived sequence, and twenty-four *Pseudomonas aeruginosa* Phage 7 (PP7) stem-loops. The PP7 stem-loops are recognized and bound by GFP-tagged PP7-Coat Protein (PCP) proteins (52), thus enabling the analysis of colocalization of chimeric TERRA molecules with telomeres detected by DNA fluorescence *in situ* hybridization (FISH) (19).

**Figure 2.**
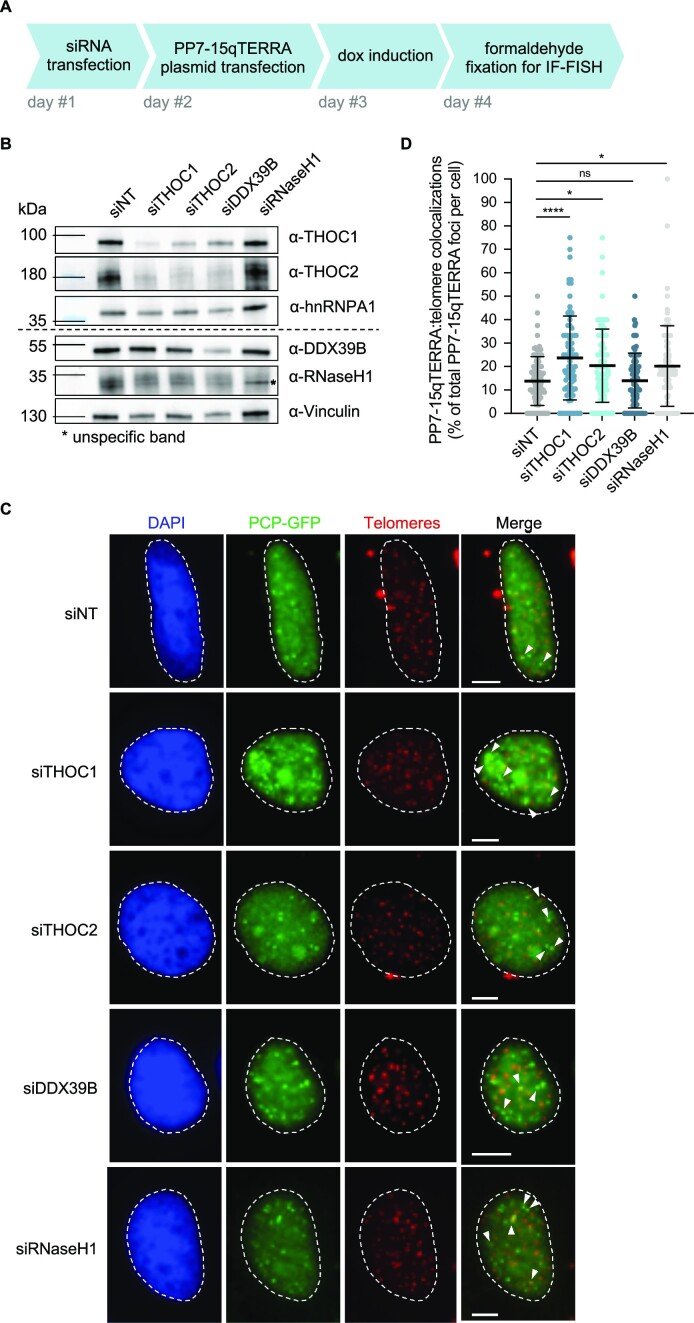
THOC subunits 1 and 2 counteract PP7-15qTERRA colocalization with telomeres. (**A**) Experimental setup: HeLa cells with 10 kb average telomere length were transfected with siRNA pools, followed by PP7-15qTERRA-expressing plasmid. Cells were fixed with formaldehyde 24 h post-PP7-15qTERRA induction and 72 h after siRNA transfection. (**B**) Western blot analysis of depletion efficiency with indicated siRNAs in HeLa cells with 10 kb average telomere length used in C and D. (**C**) Immunofluorescence of GFP-PCP (green) was employed to analyse co-localization of transiently expressed PP7-fused 15q-TERRA transcripts with telomeres (red) identified by fluorescence *in situ* hybridization (FISH). Representative images are shown for indicated conditions. White dashed lines outline the nuclear region and were determined based on DAPI-staining. White arrowheads indicate co-localization of GFP-PCP-tagged TERRA with telomeric FISH signal. Scale bars indicates 5 μm. (**D**) Quantification of colocalization of GFP-PCP-tagged PP7-15qTERRA with telomeric FISH signal, as percentage of colocalization events over total GFP-PCP-tagged PP7-15qTERRA foci, per nucleus. At least 78 cells were analysed per condition, across three independent biological replicates. Horizontal line and error bars represent mean ± s.d. One-way analysis of variance (ANOVA) with Dunnett's multiple comparisons test was applied: **** *P* ≤ 0.0001, * *P* ≤ 0.05, ns indicates non significance (*P* > 0.05).

HeLa cells with an average telomere length of 10 kb were transfected with siRNAs for depletion of THOC subunits, the TREX component DDX39B or RNaseH1. Cells were then transfected with plasmids allowing the expression of PP7-15qTERRA upon 24h doxycycline induction (Figure 2A). Notably, PP7-15qTERRA RNA molecules expressed from plasmids were found to colocalize more often with telomeres when THOC subunits 1 or 2, or RNaseH1 were depleted (Figure 2B–D). This indicates, unexpectedly, that THOC has the ability to impact PP7-15qTERRA associations with telomeres which are occurring post-transcriptionally, *in trans*. Also, in cells with shorter average telomere length (ca. 3 kb), where PP7-15qTERRA was previously shown to be recruited with higher frequency to telomeres (19), a further increase in colocalization with telomeres was observed when THOC subunits and the TREX component DDX39B were downregulated (Supplementary Figure S2A and B).

To ensure that increased colocalization prompted by loss of THOC did not stem from an increase in the nuclear PP7-15qTERRA levels, we collected total RNA from cells (10 kb average telomere length) transfected with the PP7-15qTERRA-coding plasmid and where THOC1 and -2, or RNaseH1 were downregulated (Supplementary Figure S3A), and quantified TERRA by RNA dot blot (Supplementary Figure S3B and C). While depletion of THOC1 or RNaseH1 did not disturb endogenous plus ectopically expressed TERRA levels, THOC2 depletion led to a slight reduction in UUAGGG-containing RNA levels (Supplementary Figure S3B and C). This result was accompanied by a subtle reduction in the total number of PP7-15qTERRA foci detected per nucleus (Supplementary Figure S3D). While this may reveal a different requirement of THOC by endogenous and ectopically expressed TERRA species, we consider that this should not significantly affect the analysis of TERRA association with telomeres, given that it is quantified as percentage of colocalizing TERRA foci with telomeric FISH signal, over total TERRA foci detected per nucleus. In addition, a similar effect in PP7-15qTERRA telomeric colocalizations is observed upon depletion of THOC1—which did not result in any perceivable changes in ectopically-expressed TERRA levels (Supplementary Figure S3B and C).

### The THOC complex counteracts R-loop-mediated PP7-15qTERRA associations with telomeres formed *in trans*

**Figure 3.**
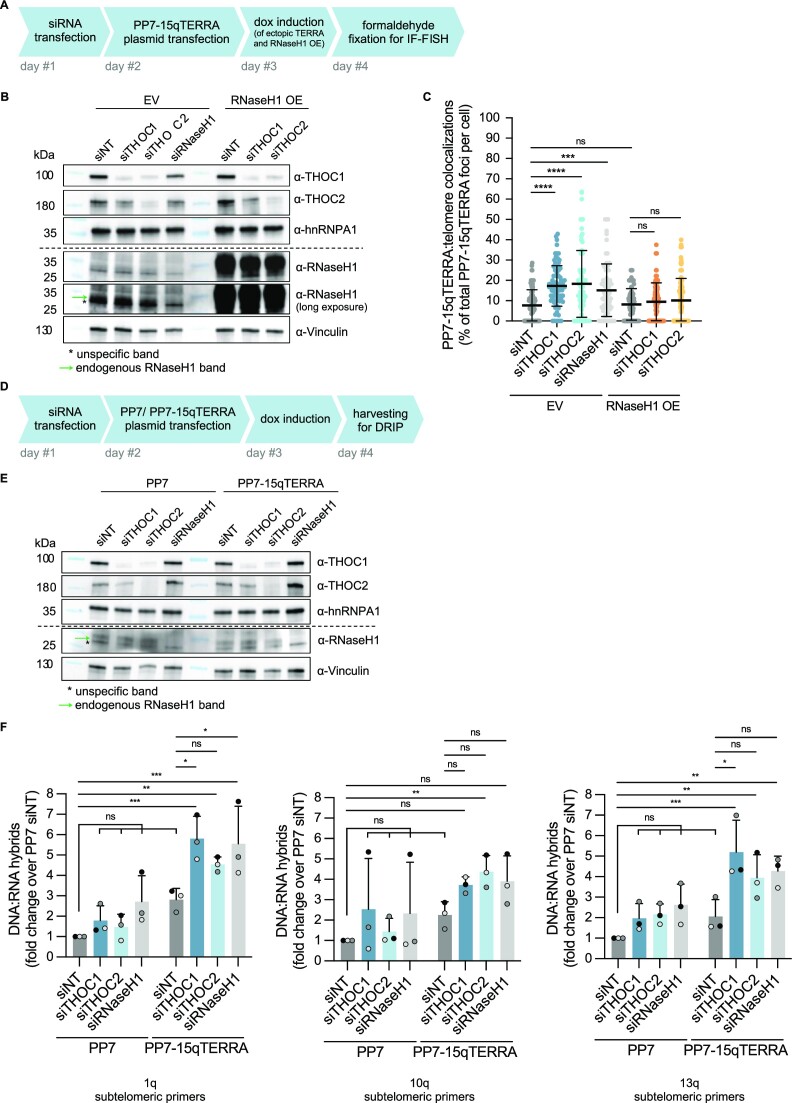
THOC counteracts TERRA R-loops formed *in trans*, post-transcriptionally. (**A**) Experimental setup: HeLa cells with 10 kb average telomere length were generated by lentivirus-transduction for doxycycline-inducible overexpression of RNaseH1-Myc-6xHis (designated RNaseH1 OE) or respective empty vector (EV) control. Cells were transfected with siRNA pools followed by PP7-15qTERRA-expressing plasmid. Cells were fixed with formaldehyde 24 h post PP7-15qTERRA induction and 72 h after siRNA transfection. (**B**) Western blot analysis of depletion efficiency with indicated siRNAs in HeLa cells overexpressing RNaseH1 or in control cells used in C. (**C**) Quantification of colocalization of GFP-PCP-tagged PP7-15qTERRA with telomeric FISH signal, as percentage of colocalization events over total GFP-PCP-tagged PP7-15qTERRA foci, per nucleus. At least 74 cells were analysed per condition, across three independent biological replicates. Horizontal line and error bars represent mean ± s.d. Two-way analysis of variance (ANOVA) with Tukey's multiple comparisons test was applied: **** *P* ≤ 0.0001, *** *P* ≤ 0.001, ns indicates non significance (*P* > 0.05). (**D**) Experimental setup: HeLa cells with 10 kb average telomere length were transfected with siRNA pools, followed by PP7- or PP7-15qTERRA-expressing plasmids. Cells were collected for DNA:RNA immunoprecipitation (DRIP) 72 h following siRNA transfection. (**E**) Western blot analysis of depletion efficiency with indicated siRNAs in HeLa cells with 10 kb average telomere length used in F. (**F**) DRIP assay using anti-DNA:RNA hybrid S9.6 antibody was performed in extracts from HeLa cells with average 10 kb telomere length. Immunoprecipitates and input samples were treated with RNase (DNase-free), purified and analysed by qPCR with primer sets amplifying 1q, 10q or 13q subtelomeric DNA. Data are mean fold change over PP7 siNT ± s.d. Two-way analysis of variance (ANOVA) with Dunnett's multiple comparisons test was applied: *** *P* ≤ 0.001, ** *P* ≤ 0.01, * *P* ≤ 0.05, ns indicates non significance (*P* > 0.05).

Aiming to determine the nature of PP7-15qTERRA associations with telomeres, prompted by loss of THOC subunits, we generated HeLa cells with inducible RNaseH1 overexpression by lentiviral transduction. In this experimental setup, PP7-15qTERRA expression is induced by doxycycline alongside with RNaseH1 overexpression (Figure 3A and B). Interestingly, we observed that PP7-15qTERRA:telomere colocalizations promoted by THOC deficiency are sensitive to RNaseH1 overexpression, being reduced to near basal levels (Figure 3C; Supplementary Figure S3E). This suggests that THOC counteracts associations formed *in trans*, which take the form of direct DNA:RNA hybrids. Additionally, in cells with shorter telomeres, RNaseH1 overexpression could rescue the telomeric colocalizations induced by THOC siRNA transfection to the basal levels observed with a non-targeting siRNA (Supplementary Figure S4A and B). However, upon RNaseH1 overexpression, colocalization levels in these cells could not be reduced to those observed in cells with longer telomere length, indicating that not all associations occur through direct DNA:RNA base-pairing.

To corroborate the formation of R-loop structures at telomeres upon expression of transgenic TERRA we employed DRIP (Figure 3D-F). In this experiment we analyzed the pulled down DNA component of DNA:RNA hybrids by qPCR, using subtelomeric-specific primers residing in sequences adjacent to the telomeric TTAGGG repetitive tract, namely at 1q, 10q and 13q subtelomeres. This ensures that R-loops formed at chromosome ends are being detected, as opposed to those which may form at the repetitive tract of plasmids from which PP7-15qTERRA RNAs are expressed (which would likely be detected when analysing DRIP samples by DNA dot blot with a probe targeting telomeric repeats). While R-loops were only slightly (non-significantly) increased upon THOC depletion under endogenous TERRA expression (PP7-control expression), as well as upon TERRA overexpression (combined with control siRNA transfection), a statistically significant increase of telomeric R-loops was detected when TERRA overexpression was combined with depletion of THOC subunits (Figure 3F). It is unclear if the slight differences at 1q, 10q and 13q telomeres are meaningful. However, the results clearly support the occurrence of TERRA-telomere associations induced by loss of THOC subunits 1 or 2 (Figure 3C). This indicates that THOC has the capacity to impact not only R-loops which are formed co-transcriptionally, but also those which form post-transcriptionally *in trans*.

### The THO complex occupancy at the telomeric tract is increased upon loss of RNaseH1

**Figure 4.**
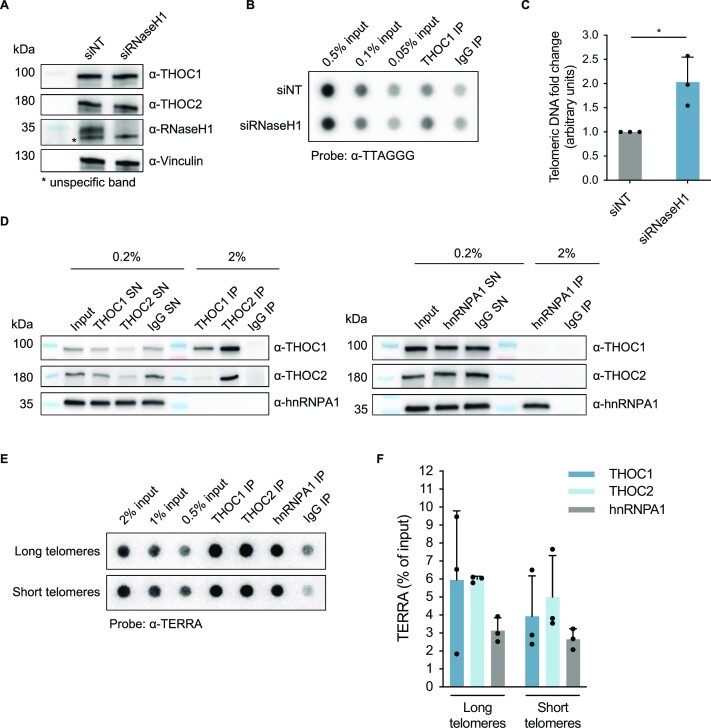
THOC1 occupancy at the telomeric tract is increased upon R-loop accumulation following RNaseH1 depletion; THOC associates with endogenous nucleoplasmic TERRA. (**A**) Western blot analysis of depletion efficiency with siRNaseH1 in HeLa cells with average 10 kb telomere length used in B and C. (**B**) Chromatin-immunoprecipitation (ChIP) assay using anti-THOC1 antibody was performed in extracts from HeLa cells with average 10 kb telomere length, transfected with siRNA to deplete RNaseH1 or control non-targeting siRNA (siNT). Samples were analysed by DNA dot blot probed with a ^32^P-radiolabelled [CCCTAA]_3_ probe. (**C**) Quantification of immunoprecipitated telomeric DNA (as in B), as fold change over siNT. The signal corresponding to immunoprecipitates using IgG antibody was subtracted from corresponding test immunoprecipitation signals as background. Data represent mean ± s.d., from three independent biological replicates. Two-tailed ratio paired t-test was applied: * *P* ≤ 0.05. (**D**) Western blot was used to evaluate the efficiency of immunoprecipitation of THOC1 and THOC2 (left hand side panel), and hnRNPA1 (right hand side panel). Samples obtained from RNA-IP with HeLa cells with average 10 kb telomere length were used in shown blots as an example. (**E**) Native RNA-IP assay using anti-THOC1, anti-THOC2 and anti-hnRNPA1 antibodies was performed in extracts from HeLa cells with long (average 10 kb, top) or short (average 3 kb, bottom) telomere length. Samples were analysed by RNA dot blot probed with a ^32^P-radiolabelled [CCCTAA]_3_ probe. (**F**) Quantification of immunoprecipitated TERRA (as in E), as percentage of input. The signal corresponding to immunoprecipitates using IgG antibody was subtracted from corresponding test immunoprecipitation signals as background. Data represent mean ± s.d., from three independent biological replicates.

The THO complex was previously found to be part of the human telomeric proteome (41). Thus, we wished to elucidate the basis of THOC recruitment to telomeres, in order to better understand the mechanism behind the observed effect in counteracting telomeric R-loops. For that end, we depleted RNaseH1 (Figure 4A)—which subtly elevates telomeric R-loop levels at telomeres (Figure 3F)—and evaluated THOC1 occupancy at telomeres by chromatin immunoprecipitation (ChIP) with an antibody against this THOC subunit. Pulled-down DNA was analyzed by DNA dot blot hybridization with a C-rich telomeric probe (Figure 4B). Interestingly, THOC1 occupancy at telomeres showed a near 2-fold increase when RNaseH1 was depleted, compared to control cells (Figure 4C). This suggests that upon R-loop accumulation at the telomeric tract, THOC is recruited to contribute to their resolution. Thus, THOC may assist the transcriptional machinery during elongation, and it may contribute to the removal of this topological obstacle from DNA also post-transcriptionally, in a timely manner, to prevent encounters with the replication machinery.

### Endogenous nucleoplasmic TERRA RNA associates with the THO complex

THOC is a known RNA-binding complex (32,53). To determine whether THOC subunits associate with TERRA, we carried out native RNA immunoprecipitation (RNA-IP) with antibodies against THOC subunits 1 and 2 (Figure 4D; Supplementary Figure S5A). As a control, hnRNPA1 was also immunoprecipitated (Figure 4D; Supplementary Figure S5A), since it is a known TERRA-binding protein (54). Remarkably, while THOC1 is specifically immunoprecipitated by anti-THOC1 antibody, both subunits THOC1 and -2 are immunoprecipitated by anti-THOC2 antibody. Pulled-down RNA was then examined by RNA dot blot, probed with a ^32^P-radiolabeled [CCCTAA]_3_ probe (Figure 4E). These experiments indicated that as hnRNPA1, both THOC1 and THOC2 associate with endogenous nucleoplasmic TERRA in nuclear extracts, irrespective of telomere length (Figure 4E and F). Conversely, the very abundant 18S rRNA was not significantly bound by THOC (Supplementary Figure S5B and C). In parallel, we performed RNA-IP experiments in Hek293E cells with anti-THOC1 and anti-THOC2 antibodies. Also in these experiments TERRA was detected in the immunoprecipitates. Furthermore, partial depletion of THOC subunits 1 and 2 with shRNAs (Supplementary Figure S6A and B) significantly reduced the amount of co-immunoprecipitated TERRA supporting the conclusion that THOC1 and THOC2 bind TERRA (Supplementary Figure S6C-G). Finally, we observed binding of TERRA to ectopically-expressed 3xHA-tagged THOC1, as assessed through RNA-IP with anti-HA antibodies, while with empty vector cells the anti-HA antibodies did not pull down TERRA (Supplementary Figure S7). Altogether, these results confirm the physical interaction between THOC and TERRA.

### The THO complex counteracts TERRA R-loop-mediated telomeric fragility

**Figure 5.**
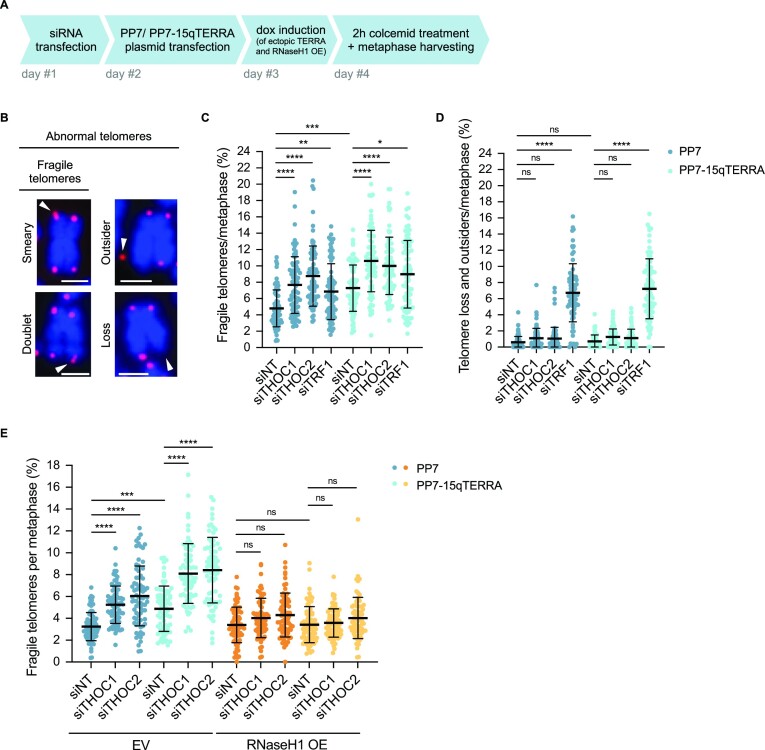
THOC prevents telomeric fragility induced by TERRA R-loops. (**A**) Experimental setup: HeLa cells with 10 kb average telomere length were transfected with siRNA pools, followed by PP7- or PP7-15qTERRA-expressing plasmids. Samples were collected after 2 h demecolcine treatment for enrichment of cells in metaphase. (**B**) Telomeric FISH on metaphase spreads of HeLa cells stained with Cy3-[CCCTAA]_3_ FISH probe (red) and DAPI (blue). White arrowheads indicate fragile telomeres—either with a smeary FISH signal (top left) or multiple FISH signals (bottom left)—or outsider telomere (top right), or telomere loss (bottom right), per single chromosome arm end. Scale bars indicate 2 μm. (C, D) Quantification of fragile telomeres (**C**), or telomere loss and outsiders (**D**), as percentage of events per metaphase spread. At least 76 metaphase spreads were analysed per condition, across three independent biological replicates. Horizontal line and error bars represent mean ± s.d. Two-way analysis of variance (ANOVA) with Tukey's multiple comparisons test was applied: **** *P* ≤ 0.0001, *** *P* ≤ 0.001, ** *P* ≤ 0.01, * *P* ≤ 0.05, ns indicates non significance (*P* > 0.05). (**E**) Quantification of fragile telomeres, as percentage of events per metaphase spread. Cells were lentivirus-transduced for doxycycline-inducible overexpression of RNaseH1-Myc-6xHis (designated RNaseH1 OE) or respective empty vector (EV) control. At least 71 metaphase spreads were analysed per condition, across three independent biological replicates. Horizontal line and error bars represent mean ± s.d. Two-way analysis of variance (ANOVA) with Tukey's multiple comparisons test was applied: **** *P* ≤ 0.0001, *** *P* ≤ 0.001, ns indicates non-significance (*P* > 0.05).

Telomeres are difficult to replicate regions, given their repetitive sequence and the multiple topological hurdles encountered at the telomeric tract. These include G-quadruplex (G4) structures, that can form on the telomeric G-rich strand which serves as a template for lagging strand DNA synthesis, as well as R-loops, which can form on the telomeric C-rich strand upon base pairing with TERRA. The C-rich strand serves as a template for leading strand DNA synthesis (12,17,18,55). Replication-dependent defects at telomeres have been shown to result in a fragile telomere phenotype observed in metaphase chromosomes. This feature is thought to originate from incomplete DNA replication or partial chromatin condensation, and is manifested as smeary or multiple telomeric FISH signals per chromosome end (56) (Figure 5). Additionally, the accumulation of telomeric G-quadruplexes and R-loop structures has previously been correlated with increased telomere fragility (12,19,57–61).

We set out to investigate whether loss of THOC subunits (Figure 5A; Supplementary Figure S8A) impacts telomeric fragility. In this analysis, depletion of the shelterin component TRF1 was included as a positive control, since it has been shown to suppress telomeric fragility, namely by recruiting the G4-unwinding BLM helicase and the transcription initiation factor TFIIH, thus sustaining replication of lagging and leading strand telomeres (56,58,62,63). Importantly, depletion of THOC subunits elevated the frequency of detected fragile telomeres (Figure 5C; Supplementary Figure S8B). This effect was also observed upon PP7-15qTERRA expression, as previously described (19), and further increased when combined with THOC depletion (Figure 5C; Supplementary Figure S8B). In addition to the fragile telomere phenotype, other telomere abnormalities were quantified, including telomere loss and outsider telomeres (Figure 5B; Supplementary Figure S8B), the latter being visualized as a telomeric FISH signal detached from chromosome ends (64). However, no significant changes were detected in the amount of outsider or lost telomeres upon depletion of THOC subunits, under endogenous or ectopically expressed TERRA levels (Figure 5D; Supplementary Figure S8B). As expected, TRF1 depletion elevated the frequency of fragile telomeres. Furthermore, TRF1 depletion resulted in the accumulation of outsider telomeres (Figure 5D; Supplementary Figure S8B).

To determine the origin of this telomeric fragility phenotype, we investigated the impact of THOC in telomeric fragility in RNaseH1 overexpressing cells (Supplementary Figure S8C). In these cells, fragility levels remained unaffected by depletion of THOC subunits, even when TERRA was overexpressed (Figure 5E; Supplementary Figure S8D). This underscores the notion that the fragility phenotype prompted by loss of THOC subunits stems from telomeric R-loops, and indicates that THOC alleviates R-loop-derived telomeric fragility, thus contributing to the suppression of telomeric replication defects. Of note, abnormalities induced by loss of TRF1 could not be rescued by RNaseH1 overexpression (Supplementary Figure S8D), consistently with previously acquired data in HeLa cells (23). This suggests that different pathways are undertaken by THOC and TRF1 to prevent telomeric aberrations.

### The THO complex counteracts leading and lagging strand telomeric fragility

**Figure 6.**
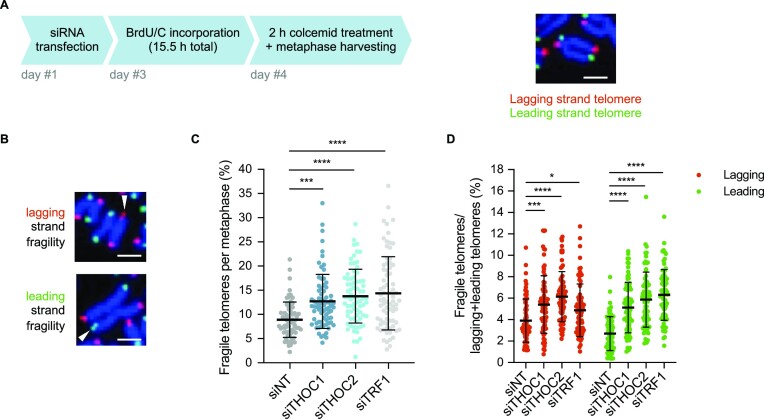
THOC prevents telomeric fragility at both lagging and leading strands. (**A**) Experimental setup: HeLa cells with 30 kb average telomere length were transfected with siRNA pools, followed by BrdU/C (3:1) incorporation for a total of 15.5 hours. Samples were collected after 2 h demecolcine treatment, for enrichment of cells in metaphase (left hand side). Representative metaphase chromosome labelled by CO-FISH, where lagging strand telomeres are labelled in red and leading strand telomeres are labelled in green (right hand side). Scale bar indicates 2 μm. (**B**) Telomeric CO-FISH on metaphase spreads of HeLa cells stained with TYE563-TeloC LNA probe (red), FAM-TeloG LNA probe (green) and DAPI (blue). White arrowheads indicate fragile telomeres. Scale bars indicate 2 μm. (**C**) Quantification of fragile telomeres in metaphase chromosomes stained by FISH, as percentage of events per metaphase spread (in HeLa cells with average 30 kb telomere length). 75 metaphase spreads were analysed per condition, across three independent biological replicates. Horizontal line and error bars represent mean ± s.d. One-way analysis of variance (ANOVA) with Dunnett's multiple comparisons test was applied: **** *P* ≤ 0.0001, *** *P* ≤ 0.001. (**D**) Quantification of lagging and leading strand fragile telomeres in metaphase chromosomes stained by chromosome orientation FISH (CO-FISH), as percentage of events per metaphase spread (sum of lagging and leading strand telomeres, in HeLa cells with average 30 kb telomere length). At least 74 metaphase spreads were analysed per condition, across three independent biological replicates. Horizontal line and error bars represent mean ± s.d. One-way analysis of variance (ANOVA) with Dunnett's multiple comparisons test was applied: **** *P* ≤ 0.0001, *** *P* ≤ 0.001, * *P* ≤ 0.05.

To better characterize the telomeric fragility observed as a consequence of R-loop accumulation, we performed chromosome-orientation telomeric FISH (CO-FISH) (65). In this experiment, BrdU and BrdC are incorporated into the nascent DNA, during a single replication cycle (Figure 6A). Upon collection of metaphase spreads, the nascent replicated strand—which incorporated BrdU/C—is degraded by UV irradiation, followed by digestion with exonuclease III. This allows specific labelling of the parental strands with different fluorescent dyes: the parental C-rich strand which serves as a template for lagging strand synthesis is labelled in red, while the G-rich strand replicated by leading strand synthesis is labeled in green (Figure 6A and B). Since optimal CO-FISH staining conditions were achieved in cells with long telomeres of approximately 30 kb length, telomeric FISH staining in metaphase spreads was repeated in these cells, so that a correlation could be established between telomeric FISH and CO-FISH stainings. Of note, basal fragility levels detected by FISH in these cells were higher (Figure 6C) than in cells with 10 kb telomeres (Figure 5C), consistent with the fact that telomere length correlates with the probability for the replication machinery to encounter obstacles, resulting in telomeric fragility. As expected, analysis of telomeric fragility by FISH upon depletion of THOC or TRF1 in cells with 30 kb average telomere length telomeres (Figure 6C; Supplementary Figure S9A) reproduced the trend observed in cells with 10 kb telomeres (Figure 5C; Supplementary Figure S8A). Interestingly, in CO-FISH experiments, depletion of THOC subunits elevated telomeric fragility at both lagging and—most prominently—leading strand telomeres (Figure 6D). Upon TRF1 depletion, telomeric fragility also increased at both strands (Figure 6D), as previously reported (23,56). In addition, TRF1 depletion resulted in increased frequency of outsider telomeres, at both strands—most notably at the telomere replicated by lagging strand synthesis (Supplementary Figure S9B-D)—a phenotype not commonly observed in mouse cells deleted of TRF1 (56,63). THOC depletion did not impact telomere loss or outsider telomere frequency (Supplementary Figure S9C, D).

### The THO complex counteracts telomeric sister chromatid exchange in U2OS cells

**Figure 7.**
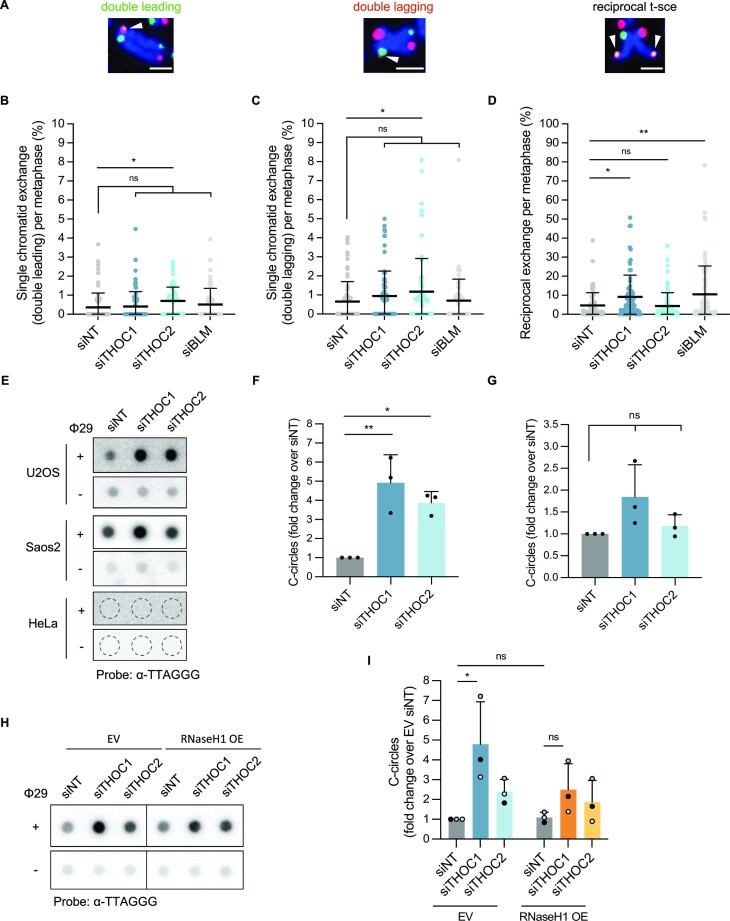
THOC counteracts telomeric sister chromatid exchange and C-circle accumulation in U2OS cells. (**A**) Telomeric CO-FISH on metaphase spreads of U2OS cells stained with TYE563-TeloC LNA probe (red), FAM-TeloG LNA probe (green) and DAPI (blue). White arrowheads indicate double leading (left hand side), double lagging (centre) or reciprocal telomeric sister-chromatid exchange (right hand side), as indicated. Scale bars indicate 2 μm. (B–D) Quantification of double leading telomeres (**B**), double lagging telomeres (**C**) or reciprocal telomeric sister-chromatid exchange (**D**), as percentage of events per metaphase spread (sum of lagging and leading strand telomeres, in U2OS cells). 75 metaphase spreads were analysed per condition, across three independent biological replicates. Horizontal line and error bars represent mean ± s.d. One-way analysis of variance (ANOVA) with Dunnett's multiple comparisons test was applied: ** *P* ≤ 0.01, * *P* ≤ 0.05, ns indicates non significance (*P* > 0.05). (**E**) Phi29-mediated C-circle assay in gDNA collected from U2OS, Saos2 or HeLa cells transfected with indicated siRNAs. Samples were analysed by DNA dot blot probed with a ^32^P-radiolabelled C-rich telomeric oligonucleotide probe (non-denatured membrane). Phi29^−^ reactions served as a control (denatured membrane). (F, G) Quantification of Phi29-mediated C-circle assay in gDNA collected from U2OS (**F**) or Saos2 (**G**) cells, as fold change over siNT. Data represent mean ± s.d., from three independent biological replicates. One-way analysis of variance (ANOVA) with Dunnett's multiple comparisons test was applied: ** *P* ≤ 0.01, * *P* ≤ 0.05, ns indicates non significance (*P* > 0.05). (**H**) Phi29-mediated C-circle assay in gDNA collected from U2OS cells transfected with indicated siRNAs and RNaseH1-Myc-6xHis-expressing construct (designated RNaseH1 OE) or respective empty vector (EV) control. Samples were analysed by DNA dot blot probed with a ^32^P-radiolabelled C-rich telomeric oligonucleotide probe (non-denatured membrane). Phi29^−^ reactions served as a control (denatured membrane). (**I**) Quantification of Phi29-mediated C-circle assay in gDNA collected from U2OS cells (as in H). Data represent mean ± s.d., from three independent biological replicates. Two-way analysis of variance (ANOVA) with Tukey's multiple comparisons test was applied: * *P* ≤ 0.05, ns indicates non significance (*P* > 0.05).

The CO-FISH protocol not only allows to examine telomeric strand-specific abnormalities, but to investigate telomeric recombination events with exchange of parental strand DNA. We analyzed both types of single sister-chromatid exchange events (single t-sce)—resulting in double leading or double lagging telomeric signals –, as well as reciprocal sister-chromatid exchange (reciprocal t-sce) (Supplementary Figure S9E; Figure 7A). Depletion of THOC subunits 1 and 2, or TRF1 in HeLa cells (Supplementary Figure S9A) led to no perceptible changes in sister-chromatid exchange events, compared to control cells (Supplementary Figure S9F). Conversely, in U2OS cells, depletion of THOC1 increased the frequency of reciprocal t-sce, while depletion of THOC2 led to a mild but significant increase of leading and lagging strand single t-sce (Figure 7B–D; Supplementary Figure S10A). Knock down of BLM helicase (Figure 7D; Supplementary Figure S10A) was used as a positive control for reciprocal t-sce in U2OS cells (66,67). These data indicate that the THO complex counteracts t-sce in ALT cells.

### The THO complex counteracts C-circle accumulation in ALT cells

We then analyzed another hallmark of ALT cells—the accumulation of extrachromosomal telomeric DNA in the form of partially single-stranded telomeric (CCCTAA)_n_ DNA circles, referred to as C-circles (46). C-circles were amplified by rolling circle amplification with Phi29 DNA polymerase and detected by non-denaturing dotblot hybridization (46). In U2OS cells, depletion of THOC1 and to a lesser extent THOC2 (Supplementary Figure S10B) led to an increase of C-circles (Figure 7E and F). This trend was also perceptible upon depletion of THOC1 or THOC2 in Saos2 cells (an ALT cell line with shorter telomere length than U2OS), although to a milder non-significant extent (Figure 7E and G; Supplementary Figure S10C). In contrast, in HeLa cells—with no detectable basal levels of C-circles (as an ALT^−^ cell line)—depletion of THOC subunits did not trigger formation of such structures (Figure 7E; Supplementary Figure S10D).

Aiming to understand whether R-loops could play a role in the C-circle accumulation observed when THOC was downregulated in ALT cells, we transiently overexpressed RNaseH1 from a plasmid in U2OS cells (Supplementary Figure S10E), and evaluated C-circle levels. RNaseH1 overexpression led to a reduction of C-circles, when combined with depletion of THOC1 (Figure 7H and I), though C-circle levels prompted by depletion of THOC2 did not exhibit clear sensitivity to RNaseH1 overexpression. Ectopically-expressed RNaseH1 protein levels were slightly reduced in siTHOC2-transfected cells, compared to non-targeting siRNA (Supplementary Figure S10E), which may have contributed to the disparity in RNAseH1-sensitivity of C-circles detected in cells depleted of THOC1 or THOC2.

Together these data point to a role of the THO complex in counteracting C-circle accumulation in ALT cells, presumably through the regulation of R-loops.

## DISCUSSION

In this paper, we explore the roles of the THO complex at human telomeres. We show that THOC negatively regulates TERRA-mediated telomeric R-loops. This was most perceptible in U2OS ALT cells, which display a stronger accumulation of telomeric DNA:RNA hybrids when compared to telomerase-positive HeLa cells (12), rendering these structures more readily detectible by DRIP. The impact of THOC depletion in telomeric R-loops does not stem from changes in TERRA transcriptional elongation, nor TERRA expression levels, since no significant changes were detected in UUAGGG-containing RNA cellular levels, nor in individual TERRA species evaluated by RT-qPCR, upon depletion of THOC.

R-loops have previously been thought to form exclusively during transcription, where the nascent RNA strand can base pair with its template DNA *in cis* (68,69). However, studies in *Saccharomyces cerevisiae* (70) and in *Arabidopsis thaliana* (71) revealed that R-loops can be formed post-transcriptionally, *in trans* at genomic regions distinct from the loci where the RNA was transcribed from. Additionally, in human cells, we have previously demonstrated that TERRA forms R-loops post-transcriptionally at telomeres, *in trans*, in a RAD51-mediated manner (19). Resorting to a published proxy for the study of the nuclear localization of TERRA (19), we found that THOC is able to counteract PP7-15qTERRA associations with chromosome ends, which are formed when TERRA is ectopically expressed from a plasmid. Particularly, we found that such associations take the form of DNA:RNA hybrids. This suggests that THOC is not only able to prevent the accumulation of co-transcriptionally formed R-loops, but also has the capacity to counteract R-loops formed post-transcriptionally, *in trans*.

**Figure 8.**
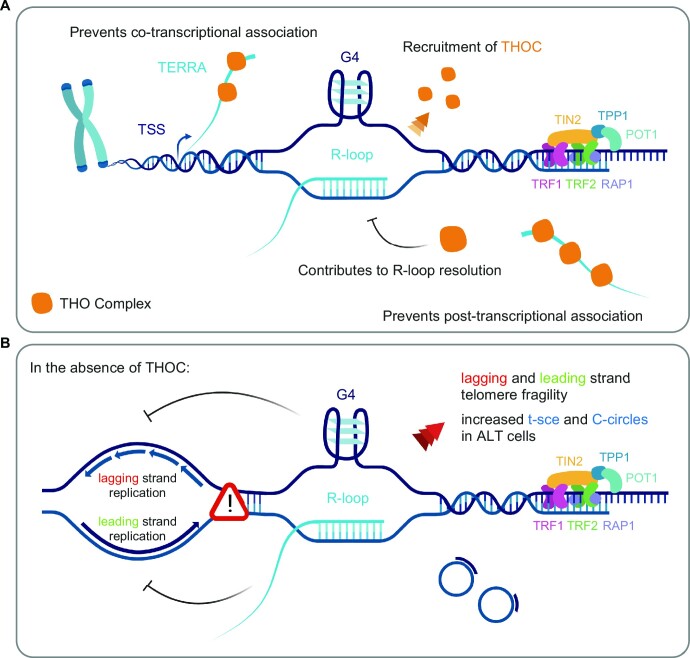
Working model for the role of THOC at human telomeres. (**A**) Interplay of THOC with TERRA and putative functions of THOC at human telomeres. (**B**) Impact of THOC deficiency in telomere fragility, telomeric sister-chromatid exchange (t-sce) and C-circle accumulation. See discussion for details. This illustration was created with BioRender.com.

A detailed mechanism through which THOC restrains DNA:RNA hybrids throughout the genome is not well elucidated. The discovery of an interplay between THOC and components of the Sin3 deacetylase complex suggested that THOC limits the formation of DNA:RNA hybrids indirectly, through the modulation of chromatin modifications (deacetylation of H3), which render the chromatin less accessible (72). In addition, DDX39B—a component of the RNA export TREX complex—was shown to directly resolve DNA:RNA hybrids through its helicase activity (36). Here, we observed a recruitment of THOC to the telomeric tract when RNaseH1 was depleted. While RNaseH1 occupancy at telomeres was shown to be much more prominent in ALT cells compared to telomerase-positive cells (12), our DRIP analysis in (telomerase-positive) HeLa cells revealed a subtle but detectable increase in telomeric R-loops upon RNaseH1 depletion. Combined, these data indicate that THOC is recruited to telomeres when R-loops accumulate (Figure 8A). This is consistent with a function of THOC in assisting with the progression of the elongating transcription machinery when R-loops accumulate (since THOC was previously found to interact with RNA Polymerase II (30)). In addition, our data suggest a role of THOC in the resolution of DNA:RNA structures formed at telomeres post-transcriptionally (Figure 8A). Along with THOC subunits (41), the TREX component DDX39B was identified at human telomeres in a mass spectrometry-based approach to identify telomeric proteins in Hek293E cells (59). We have only observed a significant change in PP7-15qTERRA localization to telomeres in HeLa cells with ca. 3 kb average telomere length, upon DDX39B depletion (Supplementary Figure S2A), but not in cells with 10 kb average telomere length depleted of DDX39B (Figure 2D). Thus, DDX39B may counteract R-loops specifically at short telomeres. It is unclear if at long telomeres THOC1/2 counteract R-loops directly or if other components are involved.

We show that THOC (subunits 1 and 2) binds nucleoplasmic endogenous TERRA in HeLa and Hek293E nuclear extracts (Figure 8A), suggesting that THOC acts directly on TERRA. Together with the physical presence of THOC at telomeres, it suggests direct functions at chromosome ends. In human cells, THOC has been found to associate with spliced, 5′ capped RNAs (32,37,38,73). Additionally, the m^6^A modification of RNA was hypothesized to contribute to THOC recruitment (74). Over 90% of human TERRA is not polyadenylated (7), and previous analysis of the 5′ subtelomeric-derived region of TERRA did not reveal any splicing events undertaken during TERRA biogenesis (5). On the other hand, TERRA contains 7-methylguanosine 5′ cap structures (7), and the m^6^A modification was recently found to be present on the subtelomeric region of TERRA (75). While the basis of THOC recruitment to TERRA remains unclear, we hypothesize that loading of THOC onto nascent TERRA and steady binding to nucleoplasmic TERRA prevents invasion into telomeric DNA, and formation of co- and post-transcriptional R-loops, respectively (Figure 8A). Furthermore, our findings are consistent with a model in which THOC remains associated with nucleoplasmic TERRA following TERRA removal from DNA:RNA hybrid structures.

R-loops formed across the genome have been shown to pose an obstacle to the transcription machinery during elongation as well as an impairment to DNA replication, resulting in accumulation of DNA damage and genomic instability (31,76–78). The fragile telomere phenotype is correlated with defects in the progression of the replication machinery at chromosome ends (56). In this study, we show that THOC counteracts telomeric fragility through its regulation of TERRA R-loops. Moreover, through CO-FISH staining of parental telomeric strands in metaphase chromosomes, we observed that telomeric fragility prompted by THOC deficiency is detected at telomeres replicated by lagging and most strikingly by leading strand synthesis. Leading strand telomeric fragility may be observed when factors that suppress telomeric R-loops are perturbed, while lagging strand telomeric fragility may result from accumulation of G4 structures, which have the propensity to form within the G-rich telomeric strand (79). Therefore, we can idealize a model in which loss of THOC results in build-up of telomeric R-loops, resulting in leading strand fragility. At the same time the displacement of the G-rich telomeric strand may stimulate formation and accumulation of G4s, which impair lagging strand replication (Figure 8B). Formation of G4s on the displaced strand by TERRA R-loops was recently proposed by Yadav and colleagues (21).

Telomeric fragility is commonly assumed to originate from replication defects and the ensuing repair processes of damaged replication forks (79). Therefore, one can hypothesize that fragility occurs as a consequence of R-loop accumulation, which generates DNA damage at telomeres. Comparison of fragility assessed by FISH with fragility (at lagging plus leading strands) analyzed by CO-FISH in HeLa cells (with the same telomere length) depleted of THOC revealed similar fragility percentages (Figure 6C and D). This suggests that fragility elicited by accumulation of R-loops upon THOC depletion does not stem from break-induced replication alone, since this repair pathway relies on conservative DNA synthesis, and its outcome would therefore not be detected by CO-FISH staining, which involves degradation of newly synthesized DNA. In mouse cells, lagging strand fragility observed in *Blm*-deficient cells was proposed to result from G4 accumulation, followed by formation of double-strand breaks repaired by break-induced replication coupled with alternative non-homologous end joining (58). Whether a similar mechanism stands behind the fragility detected by CO-FISH with our experimental setup needs to be elucidated.

TERRA levels are regulated throughout the cell cycle, reaching the lowest levels in late S phase when telomeres are replicated (7,16), presumably to reduce the frequency of R-loops which pose an obstacle to the progression of the DNA replication machinery. However, our data suggest that the repression of TERRA in S phase is not sufficient to completely eliminate telomeric R-loops, and that THOC is required to facilitate telomere replication by counteracting R-loops. Of note, the regulation of TERRA during the cell cycle is lost in ALT cancer cells (80), explaining the increased telomere replication stress that is caused by TERRA in these cells (12,14). We have previously shown that transgenic PP7-15qTERRA expressed from plasmids loses cell cycle control and associates with telomeres in S phase (20). Therefore, this system may recapitulate the replication stress observed in ALT cells.

Finally, despite the observed increase in TERRA R-loops and telomeric fragility, no changes were detected in the frequency of sister-chromatid exchange events in HeLa cells depleted of THOC subunits, compared to control siRNA. Thus, loss of THOC is not sufficient to induce this ALT-like hallmark phenotype in telomerase-positive cells. Additional events may be required to increase telomere recombination frequency, such as the modulation of the telomeric chromatin structure and telomerase inhibition (as observed upon depletion of the histone chaperones ASF1a + b in immortalized fibroblasts and HeLa cells (81). A consensually used biomarker of cells which maintain telomeres via the ALT pathway instead of telomerase is, among others, an elevated frequency of t-sce (82). In contrast to what we observed in HeLa cells, we found that THOC counteracts single and reciprocal t-sce in U2OS cells (Figure 8B), likely by regulating the association of TERRA with telomeres via R-loops. Similarly, co-depletion of NONO and SFPQ in ALT cells has been shown to result in accumulation of telomeric R-loops, as well as an increased rate of telomeric sister-chromatid exchange (61).

Finally, analysis of extrachromosomal telomeric DNA in the form of C-circles in ALT cells depleted of THOC subunits revealed a role for THOC in limiting the accumulation of C-circles (Figure 8B). Ectopic overexpression of RNaseH1 in U2OS cells reduced C-circles prompted by loss of THOC1. This suggests that R-loops mediate C-circle formation, as previously demonstrated upon dysregulation of other components involved in telomeric R-loop regulation (12,13,83,84). R-loop-mediated C-circle formation may occur through processing of R-loops (directly through excision by endonucleases such as XPF or MUS81, and self-ligation of the C-rich DNA strand) and/or processing of DNA structures resulting from R-loop accumulation (for example following break-induced replication triggered by R-loops) (84,85). Whether C-circles have direct roles in telomere maintenance serving as templates for telomeric DNA elongation, or if they simply stand as a recombination by-product is still unclear.

While TERRA was shown to be a crucial trigger/amplifier of the ALT pathway, inappropriate restriction of TERRA-induced ALT activity is thought to compromise telomere integrity (13–15). Therefore, a fine regulation of TERRA and TERRA R-loops must be attained in ALT cells. Altogether, our work demonstrates that the THO complex is one of the factors that are involved in such intricate balance at telomeres.

## Supplementary Material

gkad448_Supplemental_File

## Data Availability

The data underlying this article will be shared on reasonable request to the corresponding author.

## References

[1] de Lange T. Shelterin: the protein complex that shapes and safeguards human telomeres. Genes Dev. 2005; 19:2100–2110. 16166375 10.1101/gad.1346005

[2] Tardat M. , Déjardin J. Telomere chromatin establishment and its maintenance during mammalian development. Chromosoma. 2018; 127:3–18. 29250704 10.1007/s00412-017-0656-3 PMC5818603

[3] Azzalin C.M. , Reichenbach P., Khoriauli L., Giulotto E., Lingner J. Telomeric repeat–containing RNA and RNA surveillance factors at mammalian chromosome ends. Science. 2007; 318:798–801. 17916692 10.1126/science.1147182

[4] Nergadze S.G. , Farnung B.O., Wischnewski H., Khoriauli L., Vitelli V., Chawla R., Giulotto E., Azzalin C.M. CpG-island promoters drive transcription of human telomeres. RNA. 2009; 15:2186–2194. 19850908 10.1261/rna.1748309 PMC2779677

[5] Porro A. , Feuerhahn S., Delafontaine J., Riethman H., Rougemont J., Lingner J. Functional characterization of the TERRA transcriptome at damaged telomeres. Nat. Commun. 2014; 5:5379. 25359189 10.1038/ncomms6379 PMC4264578

[6] Feretzaki M. , Renck Nunes P., Lingner J. Expression and differential regulation of human TERRA at several chromosome ends. RNA. 2019; 25:1470–1480. 31350341 10.1261/rna.072322.119 PMC6795134

[7] Porro A. , Feuerhahn S., Reichenbach P., Lingner J. Molecular dissection of telomeric repeat-containing RNA biogenesis unveils the presence of distinct and multiple regulatory pathways. Mol. Cell. Biol. 2010; 30:4808–4817. 20713443 10.1128/MCB.00460-10 PMC2950545

[8] Arnoult N. , Van Beneden A., Decottignies A. Telomere length regulates TERRA levels through increased trimethylation of telomeric H3K9 and HP1α. Nat. Struct. Mol. Biol. 2012; 19:948–956. 22922742 10.1038/nsmb.2364

[9] Redon S. , Reichenbach P., Lingner J. The non-coding RNA TERRA is a natural ligand and direct inhibitor of human telomerase. Nucleic Acids Res. 2010; 38:5797–5806. 20460456 10.1093/nar/gkq296 PMC2943627

[10] Cusanelli E. , Romero C.A.P., Chartrand P. Telomeric noncoding RNA TERRA is induced by telomere shortening to nucleate telomerase molecules at short telomeres. Mol. Cell. 2013; 51:780–791. 24074956 10.1016/j.molcel.2013.08.029

[11] Bryan T.M. , Englezou A., Dalla-Pozza L., Dunham M.A., Reddel R.R. Evidence for an alternative mechanism for maintaining telomere length in human tumors and tumor-derived cell lines. Nat. Med. 1997; 3:1271–1274. 9359704 10.1038/nm1197-1271

[12] Arora R. , Lee Y., Wischnewski H., Brun C.M., Schwarz T., Azzalin C.M. RNaseH1 regulates TERRA-telomeric DNA hybrids and telomere maintenance in ALT tumour cells. Nat. Commun. 2014; 5:5220. 25330849 10.1038/ncomms6220 PMC4218956

[13] Silva B. , Pentz R., Figueira A.M., Arora R., Lee Y.W., Hodson C., Wischnewski H., Deans A.J., Azzalin C.M. FANCM limits ALT activity by restricting telomeric replication stress induced by deregulated BLM and R-loops. Nat. Commun. 2019; 10:2253. 31138795 10.1038/s41467-019-10179-z PMC6538666

[14] Silva B. , Arora R., Bione S., Azzalin C.M. TERRA transcription destabilizes telomere integrity to initiate break-induced replication in human ALT cells. Nat. Commun. 2021; 12:3760. 34145295 10.1038/s41467-021-24097-6 PMC8213692

[15] Silva B. , Arora R., Azzalin C.M. The alternative lengthening of telomeres mechanism jeopardizes telomere integrity if not properly restricted. Proc. Natl. Acad. Sci. U.S.A. 2022; 119:e2208669119. 36122232 10.1073/pnas.2208669119 PMC9522348

[16] Graf M. , Bonetti D., Lockhart A., Serhal K., Kellner V., Maicher A., Jolivet P., Teixeira M.T., Luke B. Telomere Length Determines TERRA and R-Loop Regulation through the Cell Cycle. Cell. 2017; 170:72–85. 28666126 10.1016/j.cell.2017.06.006

[17] Balk B. , Maicher A., Dees M., Klermund J., Luke-Glaser S., Bender K., Luke B. Telomeric RNA-DNA hybrids affect telomere-length dynamics and senescence. Nat. Struct. Mol. Biol. 2013; 20:1199–1205. 24013207 10.1038/nsmb.2662

[18] Sagie S. , Toubiana S., Hartono S.R., Katzir H., Tzur-Gilat A., Havazelet S., Francastel C., Velasco G., Chédin F., Selig S. Telomeres in ICF syndrome cells are vulnerable to DNA damage due to elevated DNA:RNA hybrids. Nat. Commun. 2017; 8:14015. 28117327 10.1038/ncomms14015 PMC5286223

[19] Feretzaki M. , Pospisilova M., Valador Fernandes R., Lunardi T., Krejci L., Lingner J. RAD51-dependent recruitment of TERRA lncRNA to telomeres through R-loops. Nature. 2020; 587:303–308. 33057192 10.1038/s41586-020-2815-6 PMC7116795

[20] Valador Fernandes R. , Feretzaki M., Lingner J. The makings of TERRA R-loops at chromosome ends. Cell Cycle. 2021; 20:1745–1759. 34432566 10.1080/15384101.2021.1962638 PMC8525998

[21] Yadav T. , Zhang J.-M., Ouyang J., Leung W., Simoneau A., Zou L. TERRA and RAD51AP1 promote alternative lengthening of telomeres through an R- to D-loop switch. Mol. Cell. 2022; 82:3985–4000. 36265486 10.1016/j.molcel.2022.09.026 PMC9637728

[22] Kaminski N. , Wondisford A.R., Kwon Y., Lynskey M.L., Bhargava R., Barroso-González J., García-Expósito L., He B., Xu M., Mellacheruvu D. et al. RAD51AP1 regulates ALT-HDR through chromatin-directed homeostasis of TERRA. Mol. Cell. 2022; 82:4001–4017. 36265488 10.1016/j.molcel.2022.09.025 PMC9713952

[23] Lee Y.W. , Arora R., Wischnewski H., Azzalin C.M. TRF1 participates in chromosome end protection by averting TRF2-dependent telomeric R loops. Nat. Struct. Mol. Biol. 2018; 25:147–153. 29358759 10.1038/s41594-017-0021-5 PMC5808845

[24] Glousker G. , Briod A.-S., Quadroni M., Lingner J. Human shelterin protein POT1 prevents severe telomere instability induced by homology-directed DNA repair. EMBO J. 2020; 39:e104500. 33073402 10.15252/embj.2020104500 PMC7705456

[25] García-Muse T. , Aguilera A. R Loops: From physiological to pathological roles. Cell. 2019; 179:604–618. 31607512 10.1016/j.cell.2019.08.055

[26] Aguilera A. , Klein H.L. HPR1, a novel yeast gene that prevents intrachromosomal excision recombination, shows carboxy-terminal homology to the *Saccharomyces cerevisiae* TOP1 gene. MOL. CELL. BIOL. 1990; 10:13. 10.1128/mcb.10.4.1439-1451.1990 PMC362246 2181275

[27] Chávez S. , Beilharz T., Rondón A.G., Erdjument-Bromage H., Tempst P., Svejstrup J.Q., Lithgow T., Aguilera A. A protein complex containing Tho2, Hpr1, Mft1 and a novel protein, Thp2, connects transcription elongation with mitotic recombination in Saccharomyces cerevisiae. EMBO J. 2000; 19:5824–5834. 11060033 10.1093/emboj/19.21.5824 PMC305808

[28] Chávez S. , García-Rubio M., Prado F., Aguilera A. Hpr1 is preferentially required for transcription of either long or G+C-rich DNA sequences in Saccharomyces cerevisiae. Mol. Cell. Biol. 2001; 21:7054–7064. 11564888 10.1128/MCB.21.20.7054-7064.2001 PMC99881

[29] Pühringer T. , Hohmann U., Fin L., Pacheco-Fiallos B., Schellhaas U., Brennecke J., Plaschka C. Structure of the human core transcription-export complex reveals a hub for multivalent interactions. Elife. 2020; 9:e61503. 33191911 10.7554/eLife.61503 PMC7744094

[30] Li Y. , Wang X., Zhang X., Goodrich D.W. Human hHpr1/p84/Thoc1 regulates transcriptional elongation and physically links RNA polymerase II and RNA processing factors. Mol. Cell. Biol. 2005; 25:4023–4033. 15870275 10.1128/MCB.25.10.4023-4033.2005 PMC1087710

[31] Domínguez-Sánchez M.S. , Barroso S., Gómez-González B., Luna R., Aguilera A. Genome instability and transcription elongation impairment in human cells depleted of THO/TREX. PLos Genet. 2011; 7:e1002386. 22144908 10.1371/journal.pgen.1002386 PMC3228816

[32] Masuda S. , Das R., Cheng H., Hurt E., Dorman N., Reed R. Recruitment of the human TREX complex to mRNA during splicing. Genes Dev. 2005; 19:1512–1517. 15998806 10.1101/gad.1302205 PMC1172058

[33] Fleckner J. , Zhang M., Valcárcel J., Green M.R. U2AF65 recruits a novel human DEAD box protein required for the U2 snRNP-branchpoint interaction. Genes Dev. 1997; 11:1864–1872. 9242493 10.1101/gad.11.14.1864

[34] Zhang M. , Green M.R. Identification and characterization of yUAP/Sub2p, a yeast homolog of the essential human pre-mRNA splicing factor hUAP56. Genes Dev. 2001; 15:30–35. 11156602 10.1101/gad.851701 PMC312605

[35] Zhou Z. , Luo M., Straesser K., Katahira J., Hurt E., Reed R. The protein Aly links pre-messenger-RNA splicing to nuclear export in metazoans. Nature. 2000; 407:401–405. 11014198 10.1038/35030160

[36] Pérez-Calero C. , Bayona-Feliu A., Xue X., Barroso S.I., Muñoz S., González-Basallote V.M., Sung P., Aguilera A. UAP56/DDX39B is a major cotranscriptional RNA–DNA helicase that unwinds harmful R loops genome-wide. Genes Dev. 2020; 34:898–912. 32439635 10.1101/gad.336024.119 PMC7328515

[37] Cheng H. , Dufu K., Lee C.-S., Hsu J.L., Dias A., Reed R. Human mRNA Export Machinery Recruited to the 5′ End of mRNA. Cell. 2006; 127:1389–1400. 17190602 10.1016/j.cell.2006.10.044

[38] Chi B. , Wang Q., Wu G., Tan M., Wang L., Shi M., Chang X., Cheng H. Aly and THO are required for assembly of the human TREX complex and association of TREX components with the spliced mRNA. Nucleic Acids Res. 2013; 41:1294–1306. 23222130 10.1093/nar/gks1188 PMC3553972

[39] Köhler A. , Hurt E. Exporting RNA from the nucleus to the cytoplasm. Nat. Rev. Mol. Cell Biol. 2007; 8:761–773. 17786152 10.1038/nrm2255

[40] Carmody S.R. , Wente S.R. mRNA nuclear export at a glance. J. Cell Sci. 2009; 122:1933–1937. 19494120 10.1242/jcs.041236 PMC2723150

[41] Grolimund L. , Aeby E., Hamelin R., Armand F., Chiappe D., Moniatte M., Lingner J. A quantitative telomeric chromatin isolation protocol identifies different telomeric states. Nat. Commun. 2013; 4:2848. 24270157 10.1038/ncomms3848

[42] Pfeiffer V. , Crittin J., Grolimund L., Lingner J. The THO complex component Thp2 counteracts telomeric R-loops and telomere shortening. EMBO J. 2013; 32:2861–2871. 24084588 10.1038/emboj.2013.217 PMC3817467

[43] Yu T.-Y. , Wang C.-Y., Lin J.-J. Depleting components of the THO complex causes increased telomere length by reducing the expression of the telomere-associated protein Rif1p. PLoS One. 2012; 7:e33498. 22448247 10.1371/journal.pone.0033498 PMC3308969

[44] Cristofari G. , Lingner J. Telomere length homeostasis requires that telomerase levels are limiting. EMBO J. 2006; 25:565–574. 16424902 10.1038/sj.emboj.7600952 PMC1383536

[45] Glousker G. , Fernandes R.V., Feretzaki M., Lingner J. Aguilera A. , Ruzov A. Detection of TERRA R-Loops at Human Telomeres. R-Loops, Methods in Molecular Biology. 2022; 2528:NY Springer US 159–171. 10.1007/978-1-0716-2477-7_11 35704191

[46] Henson J.D. , Cao Y., Huschtscha L.I., Chang A.C., Au A.Y.M., Pickett H.A., Reddel R.R. DNA C-circles are specific and quantifiable markers of alternative-lengthening-of-telomeres activity. Nat. Biotechnol. 2009; 27:1181–1185. 19935656 10.1038/nbt.1587

[47] Chávez S. , Aguilera A. The yeast *HPR1* gene has a functional role in transcriptional elongation that uncovers a novel source of genome instability. Genes Dev. 1997; 11:3459–3470. 9407037 10.1101/gad.11.24.3459 PMC316820

[48] Piruat J.I. , Aguilera A. A novel yeast gene, THO2, is involved in RNA pol II transcription and provides new evidence for transcriptional elongation-associated recombination. EMBO J. 1998; 17:4859–4872. 9707445 10.1093/emboj/17.16.4859 PMC1170815

[49] Lovejoy C.A. , Li W., Reisenweber S., Thongthip S., Bruno J., Lange T.d., De S., Petrini J.H.J., Sung P.A., Jasin M. et al. Loss of ATRX, genome instability, and an altered DNA damage response are hallmarks of the alternative lengthening of telomeres pathway. PLoS Genet. 2012; 8:e1002772. 22829774 10.1371/journal.pgen.1002772 PMC3400581

[50] Boguslawski S.J. , Smith D.E., Michalak M.A., Mickelson K.E., Yehle C.O., Patterson W.L., Carrico R.J. Characterization of monoclonal antibody to DNA · RNA and its application to immunodetection of hybrids. J. Immunol. Methods. 1986; 89:123–130. 2422282 10.1016/0022-1759(86)90040-2

[51] Cerritelli S.M. , Crouch R.J. Ribonuclease H: the enzymes in eukaryotes. FEBS J. 2009; 276:1494–1505. 19228196 10.1111/j.1742-4658.2009.06908.x PMC2746905

[52] Larson D.R. , Zenklusen D., Wu B., Chao J.A., Singer R.H. Real-time observation of transcription initiation and elongation on an endogenous yeast gene. Science. 2011; 332:475–478. 21512033 10.1126/science.1202142 PMC3152976

[53] Jimeno S. The yeast THO complex and mRNA export factors link RNA metabolism with transcription and genome instability. EMBO J. 2002; 21:3526–3535. 12093753 10.1093/emboj/cdf335 PMC126085

[54] Redon S. , Zemp I., Lingner J. A three-state model for the regulation of telomerase by TERRA and hnRNPA1. Nucleic Acids Res. 2013; 41:9117–9128. 23935072 10.1093/nar/gkt695 PMC3799450

[55] Granotier C. , Pennarun G., Riou L., Hoffschir F., Gauthier L.R., De Cian A., Gomez D., Mandine E., Riou J.-F., Mergny J.-L. et al. Preferential binding of a G-quadruplex ligand to human chromosome ends. Nucleic Acids Res. 2005; 33:4182–4190. 16052031 10.1093/nar/gki722 PMC1181860

[56] Sfeir A. , Kosiyatrakul S.T., Hockemeyer D., MacRae S.L., Karlseder J., Schildkraut C.L., de Lange T. Mammalian telomeres resemble fragile sites and require TRF1 for efficient replication. Cell. 2009; 138:90–103. 19596237 10.1016/j.cell.2009.06.021 PMC2723738

[57] Vannier J.-B. , Pavicic-Kaltenbrunner V., Petalcorin M.I.R., Ding H., Boulton S.J. RTEL1 dismantles T loops and counteracts telomeric G4-DNA to maintain telomere integrity. Cell. 2012; 149:795–806. 22579284 10.1016/j.cell.2012.03.030

[58] Yang Z. , Takai K.K., Lovejoy C.A., Lange T.d. Break-induced replication promotes fragile telomere formation. Genes Dev. 2020; 34:1392–1405. 32883681 10.1101/gad.328575.119 PMC7528700

[59] Lin C.-Y.G. , Näger A.C., Lunardi T., Vančevska A., Lossaint G., Lingner J. The human telomeric proteome during telomere replication. Nucleic Acids Res. 2021; 49:12119–12135. 34747482 10.1093/nar/gkab1015 PMC8643687

[60] Teasley D.C. , Parajuli S., Nguyen M., Moore H.R., Alspach E., Lock Y.J., Honaker Y., Saharia A., Piwnica-Worms H., Stewart S.A. Flap endonuclease 1 limits telomere fragility on the leading strand. J. Biol. Chem. 2015; 290:15133–15145. 25922071 10.1074/jbc.M115.647388 PMC4463456

[61] Petti E. , Buemi V., Zappone A., Schillaci O., Broccia P.V., Dinami R., Matteoni S., Benetti R., Schoeftner S. SFPQ and NONO suppress RNA:dNA-hybrid-related telomere instability. Nat. Commun. 2019; 10:1001. 30824709 10.1038/s41467-019-08863-1 PMC6397292

[62] Zimmermann M. , Kibe T., Kabir S., Lange T.d. TRF1 negotiates TTAGGG repeat-associated replication problems by recruiting the BLM helicase and the TPP1/POT1 repressor of ATR signaling. Genes Dev. 2014; 28:2477–2491. 25344324 10.1101/gad.251611.114 PMC4233241

[63] Yang Z. , Sharma K., de Lange T. TRF1 uses a noncanonical function of TFIIH to promote telomere replication. Genes Dev. 2022; 36:956–969. 36229075 10.1101/gad.349975.122 PMC9732906

[64] Majerska J. , Feretzaki M., Glousker G., Lingner J. Transformation-induced stress at telomeres is counteracted through changes in the telomeric proteome including SAMHD1. Life Sci. Alliance. 2018; 1:e201800121. 30456372 10.26508/lsa.201800121 PMC6238619

[65] Bailey S.M. , Goodwin E.H., Meyne J., Cornforth M.N. CO-FISH reveals inversions associated with isochromosome formation. Mutagenesis. 1996; 11:139–144. 8671729 10.1093/mutage/11.2.139

[66] Sarkar J. , Wan B., Yin J., Vallabhaneni H., Horvath K., Kulikowicz T., Bohr V.A., Zhang Y., Lei M., Liu Y. SLX4 contributes to telomere preservation and regulated processing of telomeric joint molecule intermediates. Nucleic Acids Res. 2015; 43:5912–5923. 25990736 10.1093/nar/gkv522 PMC4499145

[67] Sobinoff A.P. , Allen J.A., Neumann A.A., Yang S.F., Walsh M.E., Henson J.D., Reddel R.R., Pickett H.A. BLM and SLX4 play opposing roles in recombination-dependent replication at human telomeres. EMBO J. 2017; 36:2907–2919. 28877996 10.15252/embj.201796889 PMC5623873

[68] Crossley M.P. , Bocek M., Cimprich K.A. R-Loops as Cellular Regulators and Genomic Threats. Mol. Cell. 2019; 73:398–411. 30735654 10.1016/j.molcel.2019.01.024 PMC6402819

[69] Lafuente-Barquero J. , García-Rubio M.L., Martin-Alonso M.S., Gómez-González B., Aguilera A. Harmful DNA:RNA hybrids are formed in cis and in a Rad51-independent manner. Elife. 2020; 9:e56674. 32749214 10.7554/eLife.56674 PMC7431130

[70] Wahba L. , Gore S.K., Koshland D The homologous recombination machinery modulates the formation of RNA–DNA hybrids and associated chromosome instability. Elife. 2013; 2:e00505. 23795288 10.7554/eLife.00505 PMC3679537

[71] Ariel F. , Lucero L., Christ A., Mammarella M.F., Jegu T., Veluchamy A., Mariappan K., Latrasse D., Blein T., Liu C. et al. R-Loop mediated trans action of the APOLO long noncoding RNA. Mol. Cell. 2020; 77:1055–1065. 31952990 10.1016/j.molcel.2019.12.015

[72] Salas-Armenteros I. , Pérez-Calero C., Bayona-Feliu A., Tumini E., Luna R., Aguilera A. Human THO –Sin3A interaction reveals new mechanisms to prevent R-loops that cause genome instability. EMBO J. 2017; 36:3532–3547. 29074626 10.15252/embj.201797208 PMC5709763

[73] Merz C. , Urlaub H., Will C.L., Lührmann R. Protein composition of human mRNPs spliced in vitro and differential requirements for mRNP protein recruitment. RNA. 2007; 13:116–128. 17095540 10.1261/rna.336807 PMC1705747

[74] Lesbirel S. , Viphakone N., Parker M., Parker J., Heath C., Sudbery I., Wilson S.A. The m6A-methylase complex recruits TREX and regulates mRNA export. Sci. Rep. 2018; 8:13827. 30218090 10.1038/s41598-018-32310-8 PMC6138711

[75] Chen L. , Zhang C., Ma W., Huang J., Zhao Y., Liu H. METTL3-mediated m6A modification stabilizes TERRA and maintains telomere stability. Nucleic Acids Res. 2022; 50:11619–11634. 36399511 10.1093/nar/gkac1027 PMC9723618

[76] Huertas P. , Aguilera A. Cotranscriptionally formed DNA:RNA hybrids mediate transcription elongation impairment and transcription-associated recombination. Mol. Cell. 2003; 12:711–721. 14527416 10.1016/j.molcel.2003.08.010

[77] Wellinger R.E. , Prado F., Aguilera A. Replication fork progression is impaired by transcription in hyperrecombinant yeast cells lacking a functional THO complex. Mol. Cell. Biol. 2006; 26:3327–3334. 16581804 10.1128/MCB.26.8.3327-3334.2006 PMC1446968

[78] Gan W. , Guan Z., Liu J., Gui T., Shen K., Manley J.L., Li X. R-loop-mediated genomic instability is caused by impairment of replication fork progression. Genes Dev. 2011; 25:2041–2056. 21979917 10.1101/gad.17010011 PMC3197203

[79] Glousker G. , Lingner J. Challenging endings: how telomeres prevent fragility. Bioessays. 2021; 43:2100157. 10.1002/bies.202100157 34436787

[80] Flynn R.L. , Cox K.E., Jeitany M., Wakimoto H., Bryll A.R., Ganem N.J., Bersani F., Pineda J.R., Suvà M.L., Benes C.H. et al. Alternative lengthening of telomeres renders cancer cells hypersensitive to ATR inhibitors. Science. 2015; 347:273–277. 25593184 10.1126/science.1257216 PMC4358324

[81] O’Sullivan R.J. , Arnoult N., Lackner D.H., Oganesian L., Haggblom C., Corpet A., Almouzni G., Karlseder J. Rapid induction of alternative lengthening of telomeres by depletion of the histone chaperone ASF1. Nat. Struct. Mol. Biol. 2014; 21:167–174. 24413054 10.1038/nsmb.2754 PMC3946341

[82] Londoño-Vallejo J.A. , Der-Sarkissian H., Cazes L., Bacchetti S., Reddel R.R. Alternative lengthening of telomeres is characterized by high rates of telomeric exchange. Cancer Res. 2004; 64:2324–2327. 15059879 10.1158/0008-5472.can-03-4035

[83] Lu R. , O’Rourke J.J., Sobinoff A.P., Allen J.A.M., Nelson C.B., Tomlinson C.G., Lee M., Reddel R.R., Deans A.J., Pickett H.A. The FANCM-BLM-TOP3A-RMI complex suppresses alternative lengthening of telomeres (ALT). Nat. Commun. 2019; 10:2252. 31138797 10.1038/s41467-019-10180-6 PMC6538672

[84] Sakellariou D. , Bak S.T., Isik E., Barroso S.I., Porro A., Aguilera A., Bartek J., Janscak P., Peña-Diaz J. MutSβ regulates G4-associated telomeric R-loops to maintain telomere integrity in ALT cancer cells. Cell Rep. 2022; 39:110602. 35385755 10.1016/j.celrep.2022.110602

[85] Zhang T. , Zhang Z., Shengzhao G., Li X., Liu H., Zhao Y. Strand break-induced replication fork collapse leads to C-circles, C-overhangs and telomeric recombination. PLoS Genet. 2019; 15:e1007925. 30716077 10.1371/journal.pgen.1007925 PMC6382176

