# Supplementary material for: The THO complex counteracts TERRA R-loop-mediated telomere fragility in telomerase+ cells and telomeric recombination in ALT+ cells
Source: Nucleic Acids Res. 2023 May 29;51(13):6702–22. doi: 10.1093/nar/gkad448 (PMC10359610; doi:10.1093/nar/gkad448)
Supplement: gkad448_Supplemental_File [file gkad448_supplemental_file.pdf]

Supplementary figure 1

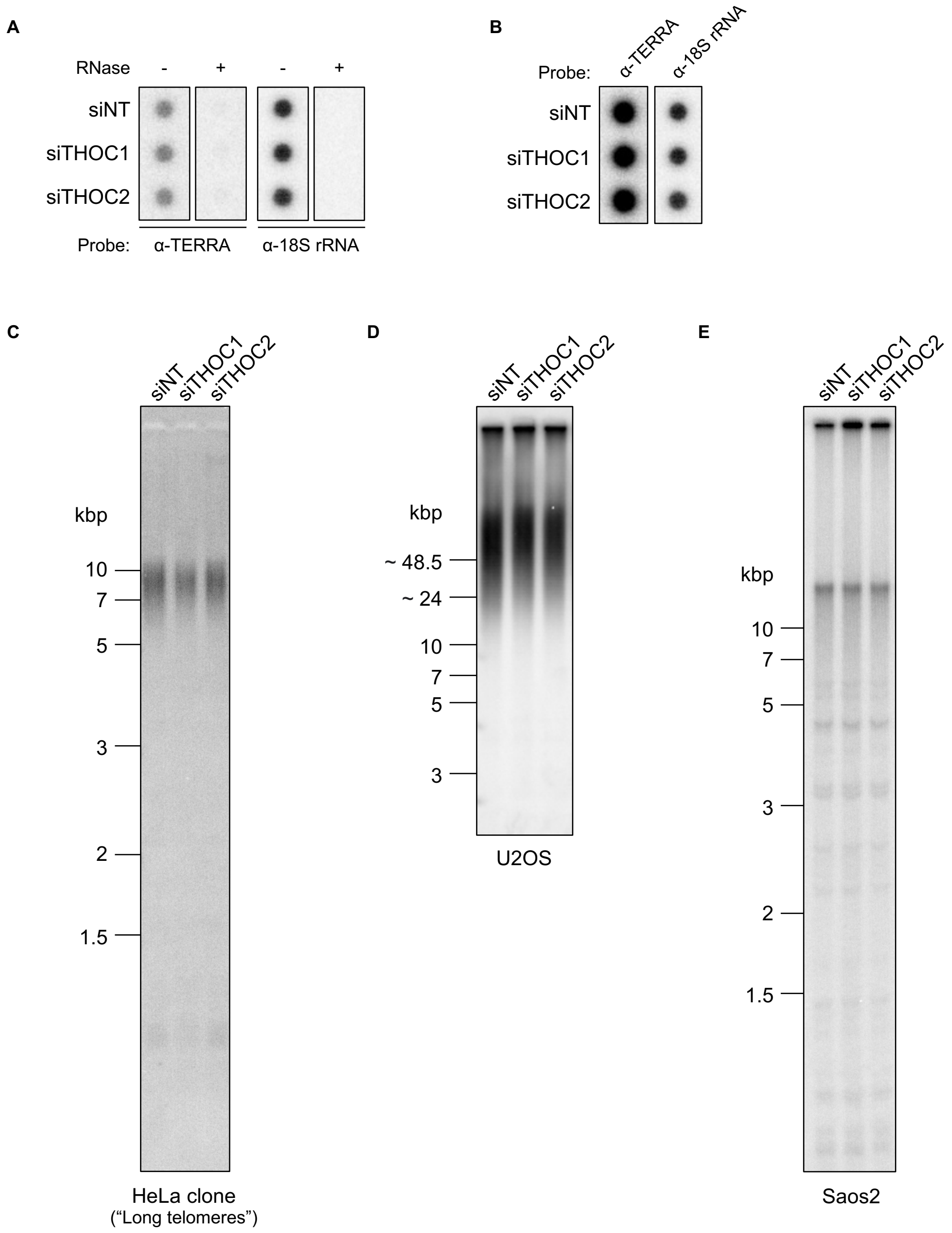

Supplementary figure 1. Depletion of THOC does not impact TERRA RNA levels nor telomere length.

A RNA dot blot analysis of TERRA or 18S rRNA levels upon depletion with indicated siRNAs in HeLa cells. 4 µg total RNA were loaded per sample. RNA samples were treated with RNase (DNase-free) as a control.

B RNA dot blot analysis of TERRA or 18S rRNA levels upon depletion with indicated siRNAs in U2OS cells. 2 µg total RNA were loaded per sample.

C Telomere restriction fragment length analysis with gDNA from HeLa cells transfected with indicated siRNAs (constant-field gel electrophoresis).

D Telomere restriction fragment length analysis with gDNA from U2OS cells transfected with indicated siRNAs (pulsed-field gel electrophoresis).

E Telomere restriction fragment length analysis with gDNA from Saos2 cells transfected with indicated siRNAs (constant-field gel electrophoresis).

Supplementary figure 2

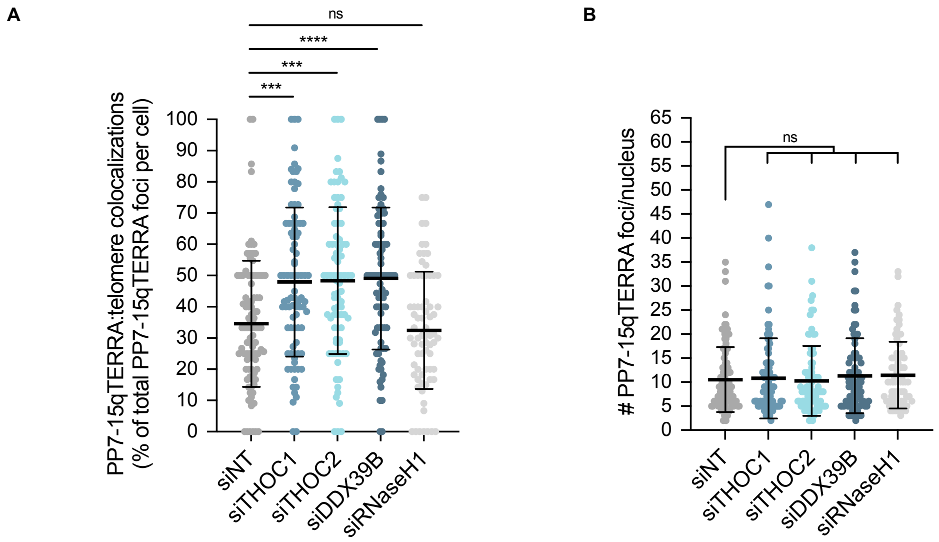

Supplementary figure 2. THOC counteracts PP7-15qTERRA colocalization with telomeres in cells with short telomeres.

A Quantification of colocalization of GFP-PCP-tagged PP7-15qTERRA with telomeric FISH signal, as percentage of colocalization events over total GFP-PCP-tagged PP7-15qTERRA foci, per nucleus, in HeLa cells with ca. 3 kb average telomere length. At least 73 cells were analysed per condition, across three independent biological replicates. Horizontal line and error bars represent mean ± s.d.. One-way analysis of variance (ANOVA) with Dunnett's multiple comparisons test was applied: **** $P \leq 0.0001$, *** $P \leq 0.001$, ns indicates non significance ($P > 0.05$).

B Number of total PP7-15qTERRA foci per nucleus, in cells of each indicated condition used for quantification of colocalization of GFP-PCP-tagged PP7-15qTERRA with telomeric FISH signal (as in A). At least 73 cells were analysed per condition, across three independent biological replicates. Horizontal line and error bars represent mean ± s.d.. One-way analysis of variance (ANOVA) with Dunnett's multiple comparisons test was applied: ns indicates non significance ($P > 0.05$).

Supplementary figure 3

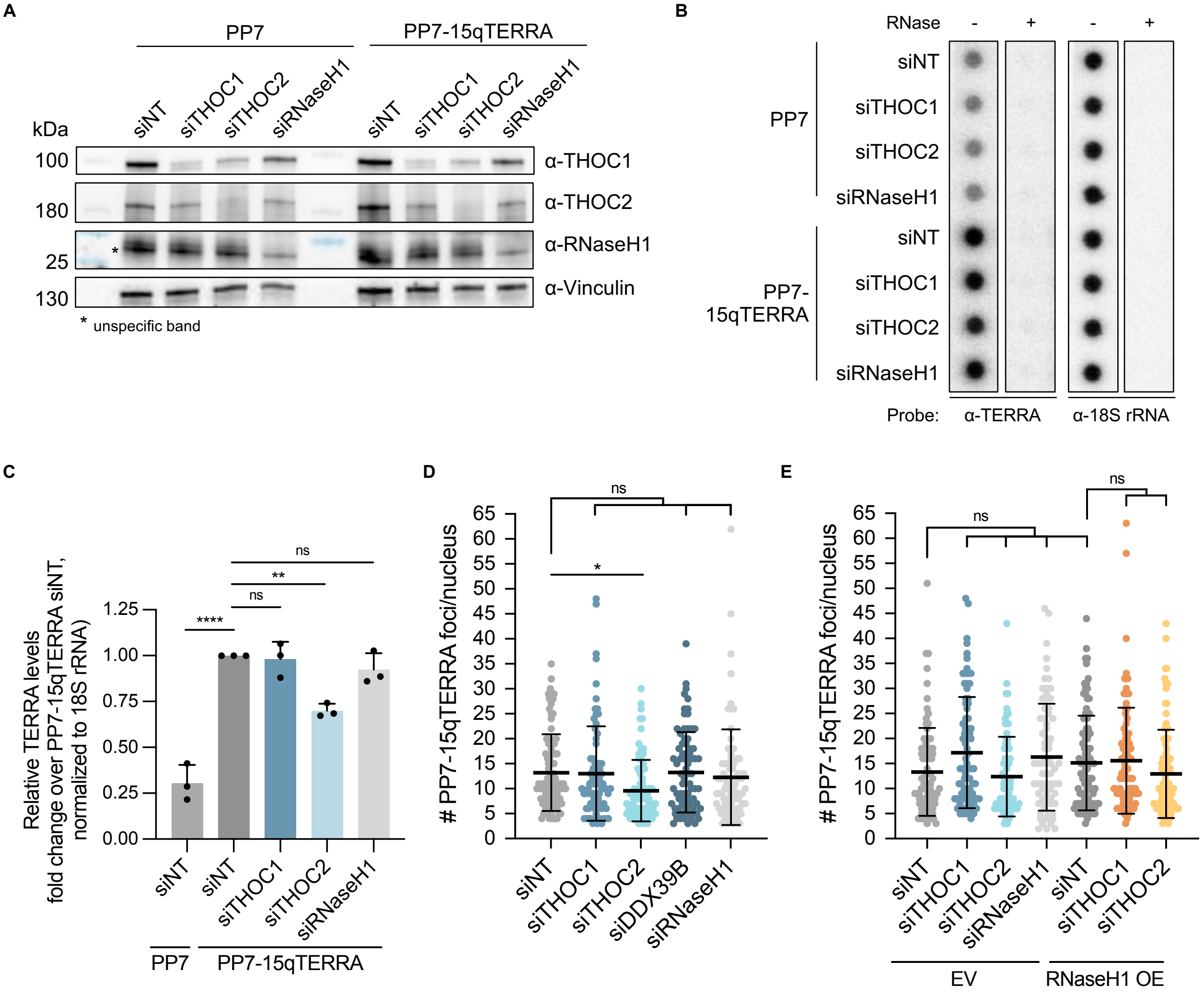

Supplementary figure 3. Analysis of PP7-15qTERRA levels by RNA dot blot and microscopy.

A Western blot analysis of depletion efficiency with indicated siRNAs in HeLa cells with average 10 kb telomere length.

B RNA dot blot analysis of TERRA or 18S rRNA levels upon depletion with indicated siRNAs in HeLa cells transfected with PP7- or PP7-15qTERRA-expressing plasmids. 4 µg total RNA were loaded per sample. RNA samples were treated with RNase (DNase-free) as a control.

C Quantification of TERRA levels (as in B), normalized to 18S rRNA levels in HeLa cells, plotted as fold change over PP7-15qTERRA siNT. Data are mean ± s.d.. One-way analysis of variance (ANOVA) with Dunnett's multiple comparisons test was applied: **** $P \leq 0.0001$, ** $P \leq 0.01$, ns indicates non-significance ($P > 0.05$).

D Number of total PP7-15qTERRA foci per nucleus, in HeLa cells with average 10 kb telomere length of each indicated condition, used for quantification of colocalization of GFP-PCP-tagged PP7-15qTERRA with telomeric FISH signal. At least 78 cells were analysed per condition, across three independent biological replicates. Horizontal line and error bars represent mean ± s.d.. One-way analysis of variance (ANOVA) with Dunnett's multiple comparisons test was applied: * $P \leq 0.05$, ns indicates non significance ($P > 0.05$).

E Number of total PP7-15qTERRA foci per nucleus, in HeLa cells with average 10 kb telomere length of each indicated condition used for quantification of colocalization of GFP-PCP-tagged PP7-15qTERRA with telomeric FISH signal. At least 74 cells were analysed per condition, across three independent biological replicates. Horizontal line and error bars represent mean ± s.d.. Two-way analysis of variance (ANOVA) with Tukey's multiple comparisons test was applied: ns indicates non significance ($P > 0.05$).

Supplementary figure 4

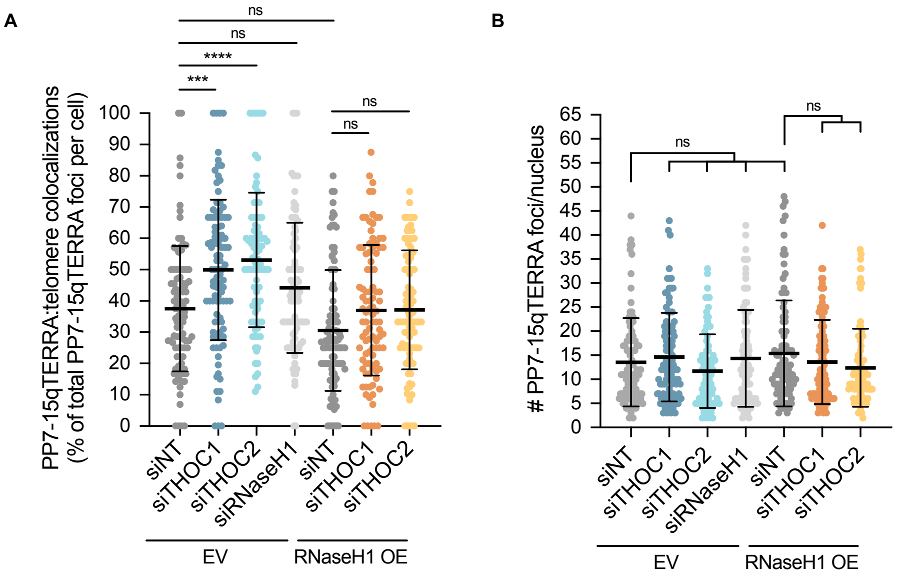

Supplementary figure 4. PP7-15qTERRA telomeric colocalizations prompted by loss of THOC are partially sensitive to RNaseH1 overexpression, in cells with short telomeres.

A Quantification of colocalization of GFP-PCP-tagged PP7-15qTERRA with telomeric FISH signal, as percentage of colocalization events over total GFP-PCP-tagged PP7-15qTERRA foci, per nucleus, in HeLa cells with 3 kb average telomere length, overexpressing RNaseH1 or in control cells, as indicated. At least 84 cells were analysed per condition, across three independent biological replicates. Horizontal line and error bars represent mean ± s.d.. One-way analysis of variance (ANOVA) with Dunnett's multiple comparisons test was applied: **** $P \leq 0.0001$, *** $P \leq 0.001$, ns indicates non significance ($P > 0.05$).

B Number of total PP7-15qTERRA foci per nucleus, in cells of each indicated condition used for quantification of colocalization of GFP-PCP-tagged PP7-15qTERRA with telomeric FISH signal (as in A). At least 84 cells were analysed per condition, across three independent biological replicates. Horizontal line and error bars represent mean ± s.d.. One-way analysis of variance (ANOVA) with Dunnett's multiple comparisons test was applied: ns indicates non significance ($P > 0.05$).

Supplementary figure 5

A

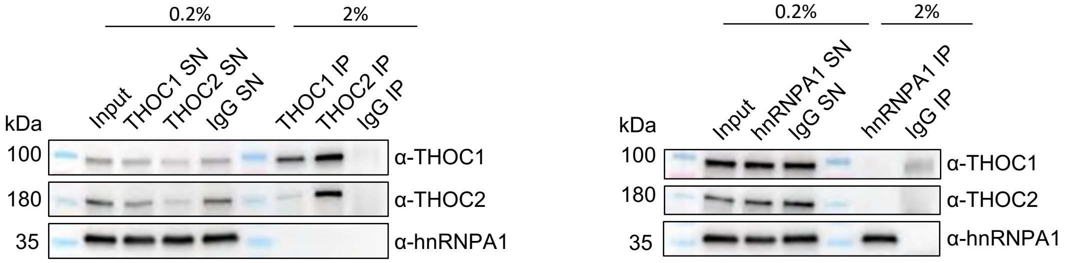

B

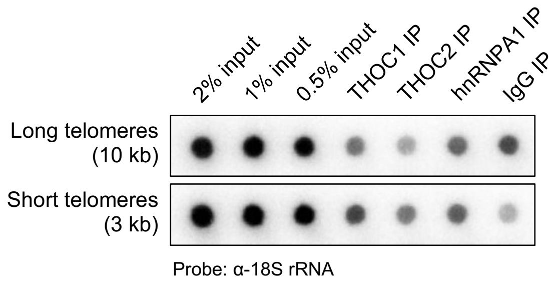

C

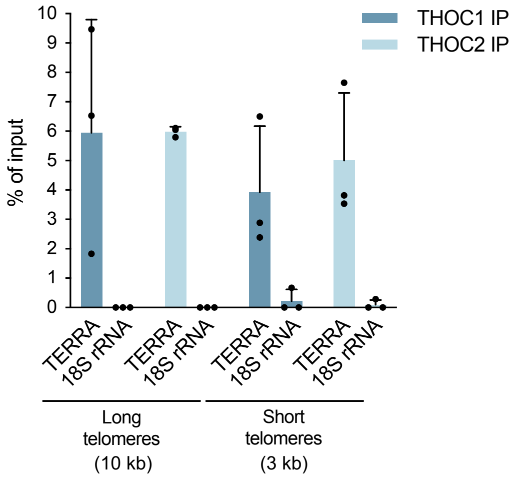

Supplementary figure 5. THOC associates with nucleoplasmic TERRA, but not the abundant 18S rRNA.

A Western blot was used to evaluate the efficiency of immunoprecipitation of THOC1 and THOC2 (left hand side panel), and hnRNPA1 (right hand side panel). Samples obtained from RNA-IP assays with HeLa cell clones with short (average 3 kb) were used in shown blots.

B RNA-IP samples from HeLa cells with long (average 10 kb, top) or short (average 3 kb, bottom) telomere length were analysed by RNA dot blot probed with a ^{32}P-radiolabelled probe complementary to the abundant 18S rRNA.

C Quantification of immunoprecipitated TERRA versus 18S rRNA (as in B), as percentage of input. The signal corresponding to immunoprecipitates using IgG antibody was subtracted from corresponding test immunoprecipitation signals as background. Data represent mean ± s.d., from three independent biological replicates, for each HeLa cell clone with long (average 10 kb) or short (average 3 kb) telomere length.

Supplementary figure 6

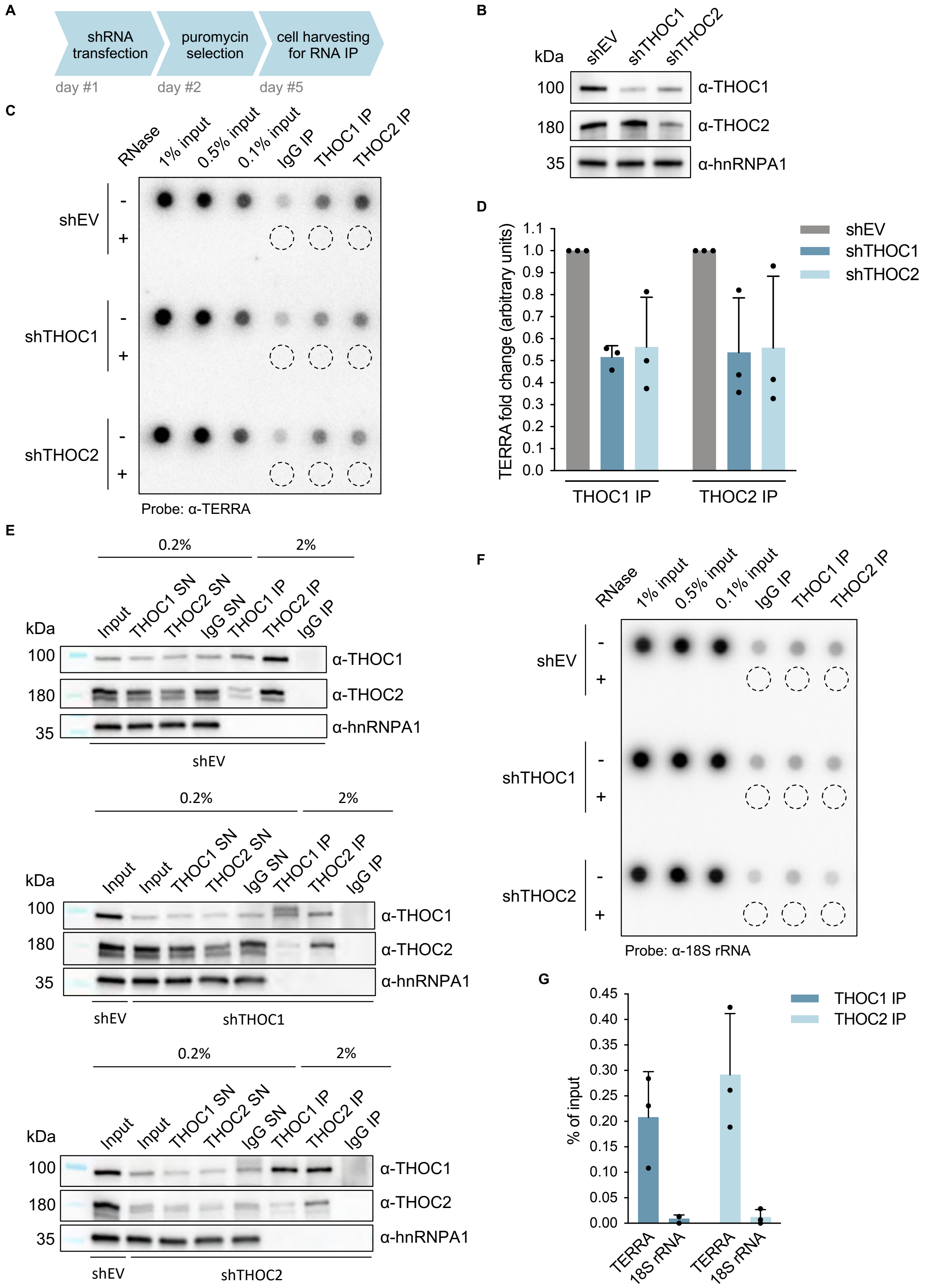

Supplementary figure 6. THOC associates with nucleoplasmic TERRA, but not the abundant 18S rRNA, in Hek293E cells.

A Experimental setup: Hek293E cells were transfected with plasmids expressing shRNAs targeting THOC1 or THOC2 (or control shRNA designated shEV). Puromycin selection of transfected cells was performed for 72 h before collecting cells for RNA-IP.

B Western blot analysis of depletion efficiency with indicated shRNAs in Hek293E cells used in C-G.

C Native RNA-IP assay using anti-THOC1 and anti-THOC2 antibodies was performed in extracts from HEK293E cells transfected with indicated shRNAs. Samples were analysed by RNA dot blot probed with a ^{32}P-radiolabelled $[CCCTAA]_3$ probe. Half of each IP sample was treated with RNase (DNase-free) as a control.

D Quantification of immunoprecipitated TERRA (as in C), as fold change over control shRNA (shEV). The signal corresponding to immunoprecipitates using IgG antibody was subtracted from corresponding test immunoprecipitation signals as background. Data represent mean ± s.d., from three independent biological replicates.

E Western blot was used to evaluate the efficiency of immunoprecipitation of THOC1 and THOC2 in samples obtained from RNA-IP assays with Hek293E cells transfected with indicated shRNAs.

F RNA-IP samples from Hek293E cells transfected with indicated shRNAs were analysed by RNA dot blot probed with a ^{32}P-radiolabelled probe complementary to the abundant 18S rRNA.

G Quantification of immunoprecipitated TERRA versus 18S rRNA (as in F), as percent of input. The signal corresponding to immunoprecipitates using IgG antibody was subtracted from corresponding test immunoprecipitation signals as background. Data represent mean ± s.d., from three independent biological replicates with Hek293E transfected with shEV.

Supplementary figure 7

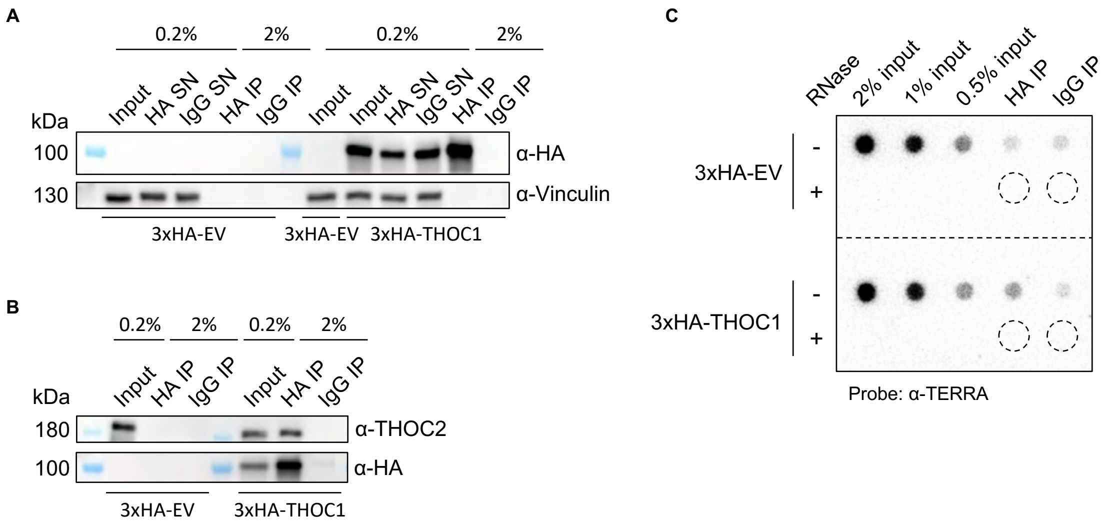

Supplementary figure 7. HA-tagged THOC1 associates with nucleoplasmic TERRA, in Hek293E cells.

A Western blot was used to evaluate the efficiency of immunoprecipitation of HA-tagged THOC1 in samples obtained from RNA-IP assays using anti-HA antibody in extracts from Hek293E cells transfected with indicated constructs.

B Western blot was used to evaluate the co-immunoprecipitation of THOC2 in samples obtained from RNA-IP assays using anti-HA antibody in extracts from Hek293E cells transfected with indicated constructs.

C Native RNA-IP assay using anti-HA antibody was performed in extracts from Hek293E cells transfected with indicated constructs. Samples were analysed by RNA dot blot probed with a ^{32}P-radiolabelled $[CCCTAA]_3$ probe. Half of each IP sample was treated with RNase (DNase-free) as a control.

Supplementary figure 8

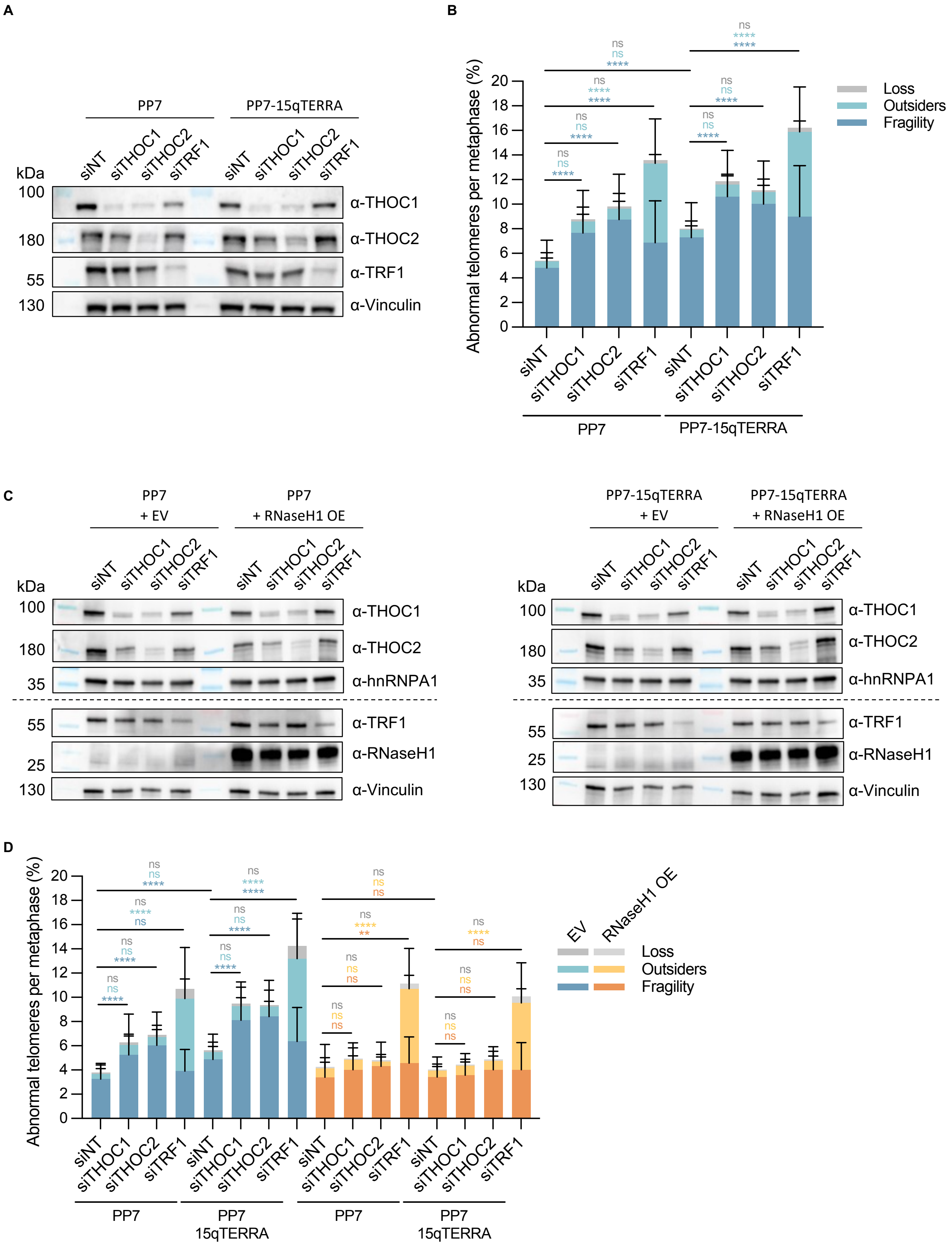

Supplementary figure 8. THOC prevents telomeric fragility induced by TERRA R-loops, but has no detectable effect in telomere loss or outsider telomeres.

A Western blot analysis of indicated proteins in lysates collected from HeLa cells with 10 kb average telomere length, transfected with specified siRNAs and with plasmids for expression of PP7 or PP7-15qTERRA transcripts, used in B.

B Quantification of telomeric abnormalities – fragile telomeres, outsider telomeres and telomeric signal loss –, plotted as percentage of events per metaphase spread. At least 76 metaphase spreads were analysed per condition, across three independent biological replicates. Data are mean ± s.d.. Two-way analysis of variance (ANOVA) with Tukey's multiple comparisons test was applied: **** $P \leq 0.0001$, ns indicates non significance ($P > 0.05$).

C Western blot analysis of indicated proteins in lysates collected from lentivirus-transduced RNaseH1 OE or control HeLa cells with 10 kb average telomere length. Cells were transfected with indicated siRNAs and with plasmids for expression of PP7 (left panel) or PP7-15qTERRA transcripts (right panel), used in D.

D Quantification of telomeric abnormalities – fragile telomeres, outsider telomeres and telomeric signal loss –, plotted as percentage of events per metaphase spread. At least 71 metaphase spreads were analysed per condition, across three independent biological replicates. Data are mean ± s.d.. Two-way analysis of variance (ANOVA) with Tukey's multiple comparisons test was applied: **** $P \leq 0.0001$, ** $P \leq 0.01$, ns indicates non significance ($P > 0.05$).

Supplementary figure 9

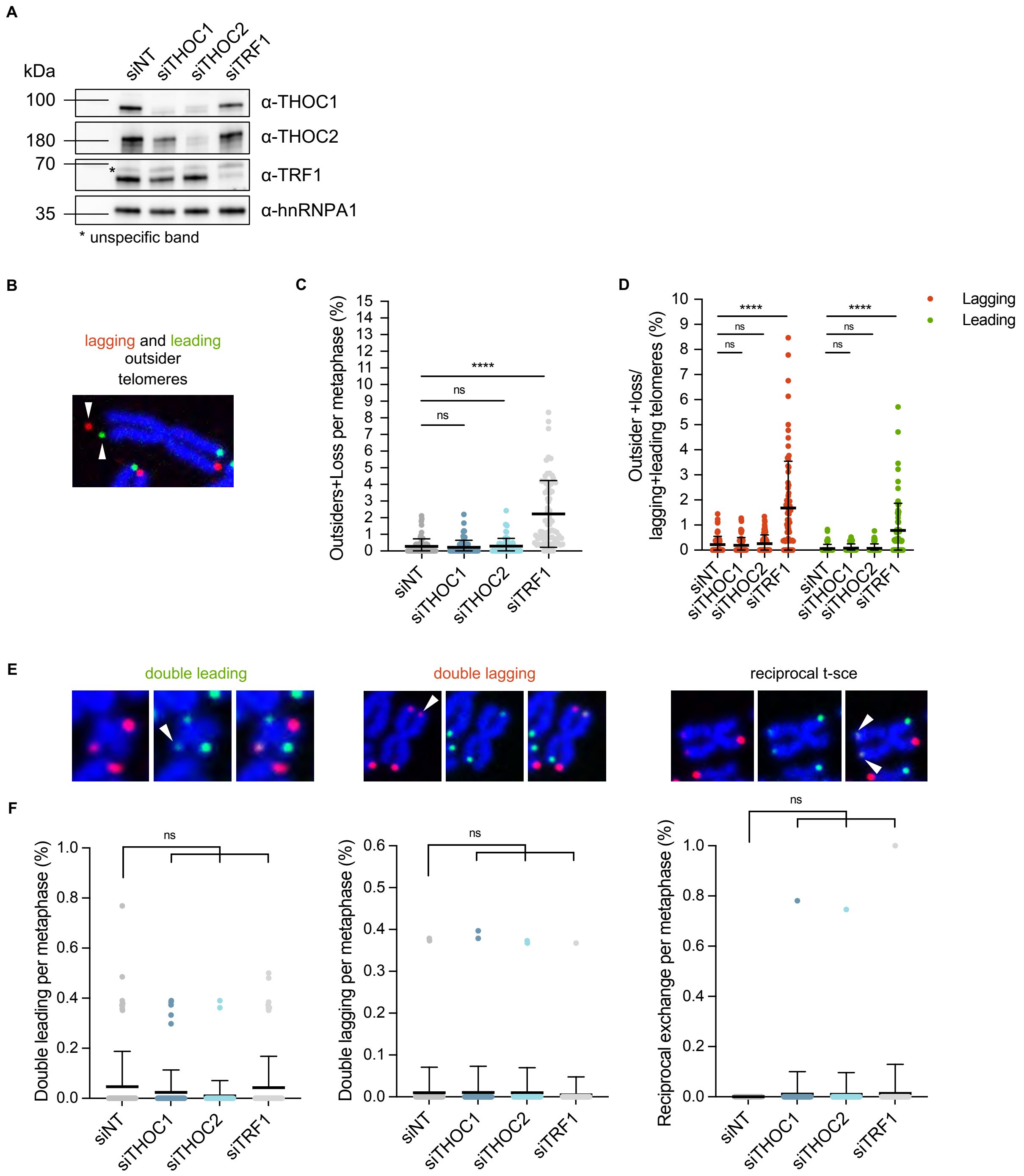

Supplementary figure 9. THOC depletion has no effect in telomeric sister-chromatid exchange events in HeLa cells.

A Western blot analysis of indicated proteins in lysates collected from HeLa cells with 30 kb average telomere length, used in FISH and CO-FISH experiments, transfected with indicated siRNAs.

B Telomeric CO-FISH on metaphase spreads of HeLa cells stained with TYE563-TeloC LNA probe (red), FAM-TeloG LNA probe (green) and DAPI (blue). White arrowheads indicate outsider telomeres.

C Quantification of outsider and lost telomeres in metaphase chromosomes stained by FISH, as percentage of events per metaphase spread (in HeLa cells with average 30 kb telomere length). 75 metaphase spreads were analysed per condition, across three independent biological replicates. Horizontal line and error bars represent mean ± s.d.. One-way analysis of variance (ANOVA) with Dunnett's multiple comparisons test was applied: **** $P \leq 0.0001$, ns indicates non significance ($P > 0.05$).

D Quantification of lagging and leading strand outsider and lost telomeres in metaphase chromosomes stained by chromosome orientation FISH (CO-FISH), as percentage of events per metaphase spread (sum of lagging and leading strand telomeres, in HeLa cells with average 30 kb telomere length). At least 74 metaphase spreads were analysed per condition, across three independent biological replicates. Horizontal line and error bars represent mean ± s.d.. One-way analysis of variance (ANOVA) with Dunnett's multiple comparisons test was applied: **** $P \leq 0.0001$, ns indicates non significance ($P > 0.05$).

E Telomeric CO-FISH on metaphase spreads of HeLa cells with 30 kb average telomere length, stained with TYE563-TeloC LNA probe (red), FAM-TeloG LNA probe (green) and DAPI (blue). White arrowheads indicate double leading telomeres (left hand side), double lagging telomeres (centre) or reciprocal telomeric sister-chromatid exchange (right hand side).

F Quantification of double leading telomeres (left hand side), double lagging telomeres (centre) or reciprocal telomeric sister-chromatid exchange (right hand side), as percentage of events per metaphase spread (sum of lagging and leading strand telomeres, in HeLa cells with average 30 kb telomere length). At least 74 metaphase spreads were analysed per condition, across three independent biological replicates. Horizontal line and error bars represent mean ± s.d.. One-way analysis of variance (ANOVA) with Dunnett's multiple comparisons test was applied: ns indicates non significance ($P > 0.05$).

Supplementary figure 10

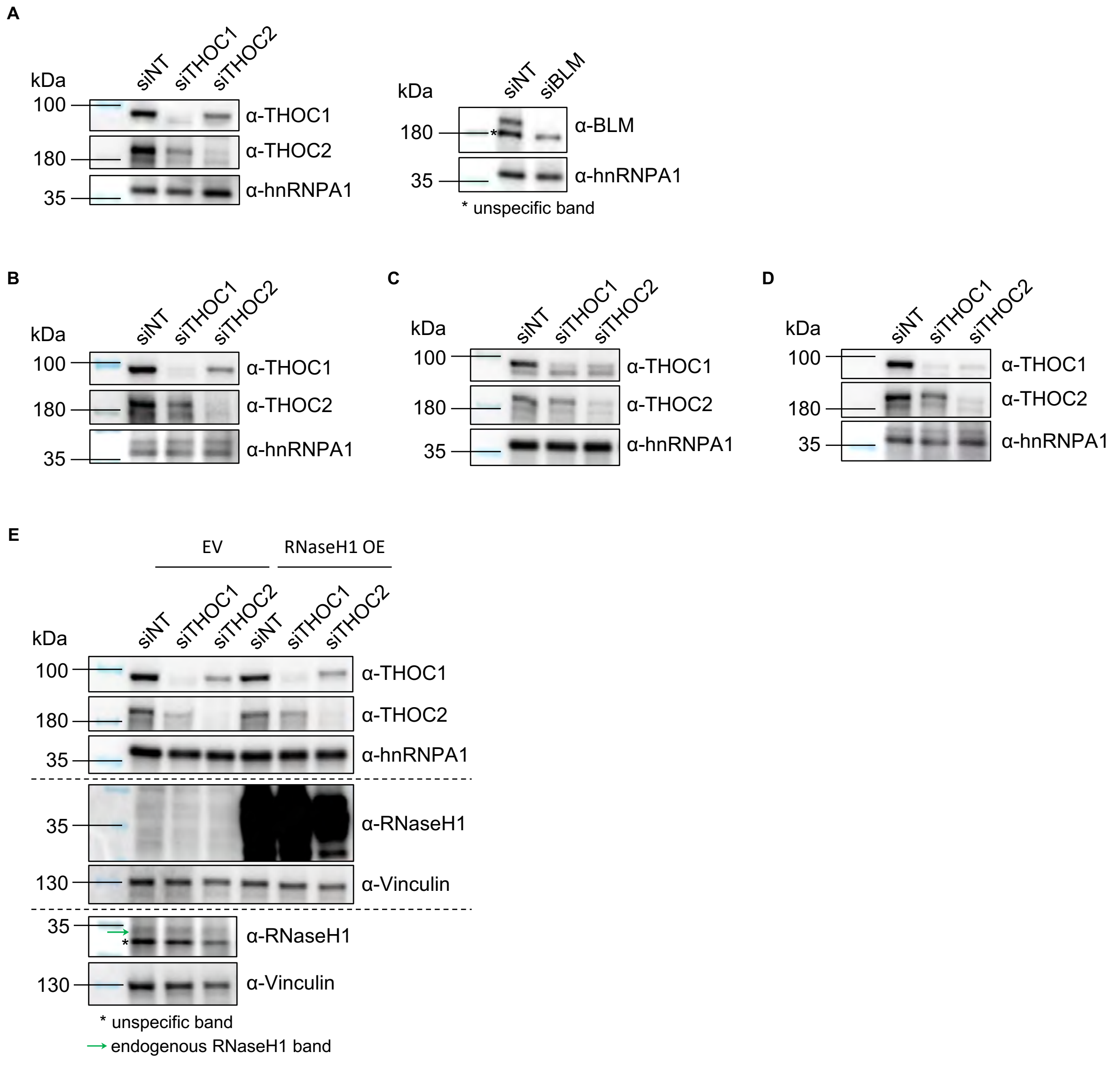

Supplementary figure 10. THOC depletions in U2OS, Saos2 and HeLa cells.

A Western blot analysis of indicated proteins in lysates collected from U2OS cells, used in CO-FISH experiments, transfected with indicated siRNAs.

B-D Western blot analysis of indicated proteins in lysates collected from U2OS (B), Saos2 (C) and HeLa (D) cells, used in C-circle assay, transfected with indicated siRNAs.

E Western blot analysis of indicated proteins in lysates collected from U2OS cells transfected with indicated siRNAs and RNaseH1-Myc-6xHis-expressing construct (designated RNaseH1 OE) or respective empty vector (EV) control, used in C-circle assay.

Supplementary table 1. siRNA sequences

siRNA	siRNA target sequences	Supplier: catalog number
siNT (non-targeting)	UAGCGACUAAACACAUCAA,<br>UAAGGCUAUGAAGAGAUAC,<br>AUGUAUUGGCCUGUAUUAG,<br>AUGAACGUGAAUUGCUCAA	Dharmacon:<br>D-001206-13
siTHOC1	GCAAUGAUCUCCUAAGAAG,<br>GAGGAGAACAUGUAUAUUU,<br>GGUCUUAACUUGCAGAGUC,<br>UAACUUUCGUCGACACAUC	Dharmacon:<br>M-019911-01
siTHOC2	GGAGAGACGUGUUCAAUAU,<br>GAAAUAAGGCUGAUCAAUU,<br>CAGCAUAGAUAUCGUCUGU,<br>AAAGAACGCCGAAGUCUGA	Dharmacon:<br>M-025006-01
siRNaseH1	GCGCAGAGCCGUAUGCAAA,<br>GAGCUAAACAAUCGGAAGA,<br>GCCAGGCCAUCCUUUAAAU,<br>GACAUUCAGUGGAUGCAUG	Dharmacon:<br>M-012595-00
siTRF1	CAAGAUAAACCUAGUGGUA,<br>GGUGAUCCAAAUUCUCAUA,<br>GGAAACUGGUCUAAAAUAC,<br>GCCAGUUGAGAACGAUAUA	Dharmacon:<br>M-010542-02
siDDX39B	ACAAUGAGCUCUUGGACUA,<br>GGAUGAAUGUGAUAAGAUG,<br>UUAGUGAGCUGCCUGAUGA,<br>UAUGAGCGCUUCUCUAAAU	Dharmacon:<br>M-003805-00
siBLM	GAGCACAUCUGUAAAUUAA,<br>GAGAAACUCACUUCAAUAA,<br>CAGGAUGGCUGUCAGGUUA,<br>CUAAAUCUGUGGAGGGUUA	Dharmacon:<br>M-007287-02

Supplementary table 2. Plasmids

Plasmid	Source	Notes
pMD2.G	Kind gift from D. Trono, EPFL	VSV-G envelope expressing plasmid, used for lentivirus production
pCMVR8.74	Kind gift from D. Trono, EPFL	Lentiviral packaging plasmid, used for lentivirus production
pTRE2_24xPP7_Puro	Feretzaki *et al*, 2020	Doxycycline inducible expression of PP7 RNA
pTRE2_24xPP7_15q subtel_90xTTAGGG_Puro	Feretzaki *et al*, 2020	Doxycycline inducible expression of PP7-15qTERRA RNA
pLenti_RNaseH1_Myc/His_Puro	Backbone: kind gift from E. Meylan, ELB	RNaseH1-Myc/His lentiviral transduction (doxycycline-inducible expression); backbone: pCW22_TREtight_Blast (selection marker exchanged with puromycin)
pcDNA6-RNAseH1-myc-His	RNaseH1 sequence amplified from construct kindly gifted by A. Straight, Stanford	Ectopic-expression of RNaseH1; backbone: pcDNA6
pshEV_Puro	Backbone: Oligoengine	Negative control shRNA; backbone: pSuper_Puro
pshTHOC1_Puro		Expression of shRNA for THOC1 depletion; backbone: pSuper_Puro
pshTHOC2_Puro		Expression of shRNA for THOC2 depletion; backbone: pSuper_Puro

Supplementary table 3. Antibodies

Antibody	Supplier: catalog number	Dilution/Amount	Application
α-THOC1	GeneTex: GTX118740	1:1,500	Western Blot
		6 μg	RNA Immunoprecipitation
		4 μg	Chromatin Immunoprecipitation
α-THOC2	Bethyl: A303-629A-T	1:10,000	Western Blot
α-THOC2	Abcam: ab129485	6 μg	RNA Immunoprecipitation
α-RNaseH1	GeneTex: GTX117624	1:1,000	Western Blot
α-TRF1	Santa Cruz: sc-6165-R	1:1,000	Western Blot
α-DDX39B	Proteintech: 14798-1-AP	1:10,000	Western Blot
α-BLM	Abcam: ab476	1:2,000	Western Blot
α-hnRNPA1	Santa Cruz: sc-32301	1:1,000	Western Blot
		6 μg	RNA Immunoprecipitation
α-Vinculin	Abcam: ab129002	1:10,000	Western Blot
α-HA	BioLegend: BGL901502	6 μg	RNA Immunoprecipitation
α-DNA:RNA hybrid (S9.6)	Kerafast: ENH001	0.1 or 0.48 μg/μg nucleic acids	DNA:RNA Immunoprecipitation
α-mouse IgG (H+L) HRP-conjugated	Promega: W4021	1:10,000	Western Blot
α-rabbit IgG (H+L) HRP-conjugated	Promega: W4011	1:10,000	Western Blot
α-GFP	*Homemade*	1:1,000	Immunofluorescence
α-rabbit IgG (H+L) CrossAdsorbed secondary antibody, Alexa Fluor 633	Thermo Fisher: A-21070	1:1,000	Immunofluorescence

Supplementary table 4. Oligonucleotide sequences

<table>
<tr><th colspan="2">Purpose</th><th>Oligonucleotide sequences</th></tr>
<tr><td colspan="2">TERRA reverse-transcription</td><td>CCCTAACCCTAACCCTAACCCTAACCCTAA</td></tr>
<tr><td colspan="2">GAPDH reverse-transcription</td><td>GCCCAATACGACCAAATCC</td></tr>
<tr><td colspan="2">1q TERRA qPCR</td><td>CAGCGTCGCAACTCAAATG, CCCTCACCCTCCATGAGTAATA</td></tr>
<tr><td colspan="2">10q TERRA qPCR</td><td>GCCTTGCCTTGGGAGAATCT, AAAGCGGGAAACGAAAAGC</td></tr>
<tr><td colspan="2">13q TERRA qPCR</td><td>GCACTTGAACCCTGCAATACAG, CCTGCGCACCGAGATTCT</td></tr>
<tr><td colspan="2">15q TERRA qPCR</td><td>TGCAACCGGGAAAGATTTTATT, GCGTGGCTTTGGGACAACT</td></tr>
<tr><td colspan="2">GAPDH qPCR</td><td>AGCCACATCGCTCAGACAC, GCCCAATACGACCAAATCC</td></tr>
<tr><td colspan="2">1q subtelomere for DRIP-qPCR</td><td>CAGCGTCGCAACTCAAATG, CCCTCACCCTCCATGAGTAATA</td></tr>
<tr><td colspan="2">10q subtelomere for DRIP-qPCR</td><td>GCATTCCTAATGCACACATGAC, TACCCGAACCTGAACCCTAA</td></tr>
<tr><td colspan="2">13q subtelomere for DRIP-qPCR</td><td>GCACTTGAACCCTGCAATACAG, CCTGCGCACCGAGATTCT</td></tr>
<tr><td rowspan="2">Generation of pLenti_RNaseH1_ Myc/His_Puro</td><td>Replacing Blast with Puro selection marker by In-Fusion cloning</td><td>TGATAAGCTTGCCACATGACCGAGTACAAGCCCAC, AGTTAAGAATACGATTCAGGCACCGGGCTTG</td></tr>
<tr><td>Insertion of RNaseH1 by In-Fusion cloning</td><td>GATCGCCTGGAGGTTAACATGAGCTGGCTTCTGTTCCT, ATCTTGGGTGGGTTAATTAATCAATGGTGATGGTGATGATGACCG</td></tr>
<tr><td colspan="2">Generation of pshTHOC1_Puro by restriction cloning</td><td>GATCCCCGCAATGATCTCCTAAGAAGTTCAAGAGACTTCTTAGGAGATCATTGC TTTTTGGAAA, AGCTTTTCCAAAAAGCAATGATCTCCTAAGAAGTCTCTTGAACTTCTTAGGAGATCATTGCGGG</td></tr>
<tr><td colspan="2">Generation of pshTHOC2_Puro by restriction cloning</td><td>GATCCCCGAAATAAGGCTGATCAATTTTCAAGAGAAATTGATCAGCCTTATTTCTTTTTGGAAA, AGCTTTTCCAAAAAGAAATAAGGCTGATCAATTTCTCTTGAAAATTGATCAGCCTTATTTCGGG</td></tr>
</table>
